# The Benthic Dinoflagellate *Coolia malayensis* (Dinophyceae) Produces an Array of Compounds with Antineoplastic Activity in Cells of Tumor Origin

**DOI:** 10.3390/md23030127

**Published:** 2025-03-14

**Authors:** Itzel B. Morales-Montesinos, Maria Yolanda Rios, Yordin D. Ocampo-Acuña, Baldomero Esquivel-Rodríguez, Celia Bustos-Brito, María del Carmen Osorio-Ramírez, Lorena M. Durán-Riveroll, Leticia González-Maya

**Affiliations:** 1 Facultad de Farmacia, Universidad Autónoma del Estado de Morelos, Av. Universidad 1001, Col. Chamilpa, Cuernavaca 62209, Mexico; itzel.moralesm@uaem.edu.mx; 2 Centro de Investigaciones Químicas, IICBA, Universidad Autónoma del Estado de Morelos, Av. Universidad 1001, Col. Chamilpa, Cuernavaca 62209, Mexico; myolanda@uaem.mx (M.Y.R.); yordin.coampoa@uaem.edu.mx (Y.D.O.-A.); 3 Instituto de Química, Universidad Nacional Autónoma de México, Circuito Exterior, Ciudad Universitaria, Mexico City 04510, Mexico; baldo@unam.mx (B.E.-R.); celia.bustos@iquimica.unam.mx (C.B.-B.); 4 Departamento de Biotecnología Marina, Centro de Investigación Científica y de Educación Superior de Ensenada, Ensenada 22860, Mexico; osoriorm@cicese.edu.mx; 5 SECIHTI-Departamento de Biotecnología Marina, Centro de Investigación Científica y de Educación, Superior de Ensenada, Ensenada 22860, Mexico

**Keywords:** antineoplastic molecules, apoptosis, benthic harmful algal blooms (bHABs), cancer research, natural products, phycotoxins

## Abstract

Among aquatic organisms, marine dinoflagellates are essential sources of bioactive metabolites. The benthic dinoflagellate *Coolia malayensis* produces metabolites that have exhibited substantial and specific cytotoxicity on cancer cells; however, isolation and identification of the purified compounds remain a challenge. This study reports *C. malayensis* biomass multi-step extraction plus chemical analyses for identifying compounds with antineoplastic activity. Through bio-directed fractionation, the cytotoxicity of extracts and fractions was tested on H1299 (lung), PC-3 (prostate), HeLa (cervical), and MCF-7 (breast) cancer cell lines. Dichloromethane (DCM) phase, hydroalcoholic (HYD) secondary extract, and methanolic (MET) extract showed cytotoxic effects on all cell lines. Active extracts and fractions were analyzed by HPLC-QTOF-MS, ^1^H, and ^13^C NMR. Cell lines H1299 and PC-3 treated with fractions F4, F7, and DCM2-AQ-Ch sub-extract showed morphological changes resembling those observed in the apoptosis control, and no signs of necrosis were observed. The selectivity of fraction F7 was above 100 μg mL^−1^ for healthy cells, while cytotoxic activity was observed in cancer cells. This fraction was identified as mostly fatty acids (FA) by NMR. Seventeen compounds with reported biological activities, such as antioxidant, analgesic, antiviral, and anticancer, were identified from *C. malayensis* extracts and fractions. Among them, the phycotoxins gambieric acid A and B, okadaic acid, and dinophysistoxin-1 were detected. Further studies are needed to reveal more significant anti-cancer potential from *C. malayensis*.

## 1. Introduction

### 1.1. The Benthic Dinoflagellate Coolia Malayensis

Benthic dinoflagellates are single-celled, photosynthetic microorganisms that primarily inhabit benthic (bottom) coastal ecosystems. They are essential components that contribute to food chains and interactions among species [1]. Benthic dinoflagellates do not create large and visible “blooms” like their planktonic counterparts but can form dense groups of cells attached to their hosts. When these dense groups are associated with harmful effects, they are called “benthic harmful algal blooms” (bHABs), though the term is not yet well defined [2]. While benthic dinoflagellates are better studied in warm tropical waters, they can be found worldwide, even in colder regions [3].

Organisms of the genus *Coolia* are marine epibenthic dinoflagellates classified within the family Ostreopsidaceae [4]. Currently, eight species have been identified, but the genus is still under review [5]. Their cells are spherical, and the size may vary among species, typically reported with measurements ranging from 23 to 50 μm in diameter [6].

The cosmopolitan species *Coolia malayensis* exhibits a wide geographical distribution, succeeding in diverse habitats from the tropics to temperate regions, including Thailand, Australia, New Zealand, and various locations within the Americas [6]. This benthic species, often found epiphytically, has been implicated in the production of toxins, with cooliatoxin (CTX) being the first such compound identified from an Australian isolate. Initially classified as a yessotoxin (YTX) analog, CTX has since been recognized as a distinct polyether ladder toxin. Furthermore, *C. malayensis* has been documented to synthesize gambierone and 44-methyl-gambierone [7].

Various studies have revealed that extracts from some species, including *C. malayensis*, exhibit mechanisms influencing mitochondrial oxidative phosphorylation and mitochondrial permeability transition (MPT) in hepatocellular carcinoma cells (HepG2); however, the isolation and identification of the compounds responsible for this activity have not yet been successful [8]. Methanolic extracts from different strains of *C. malayensis* have shown limited effects at concentrations of 25 and 50 μg mL^−1^ on HL-60 (human Caucasian promyelocytic leukemia) and RAW 264.7 (murine macrophages) cells [9]. On the other hand, crude extracts from New Zealand strains have demonstrated cytotoxic effects on HepG2 and H9c2 (rat embryonic heart tissue myoblasts) cells [8].

Many dinoflagellates produce a wide variety of complex and often structurally novel secondary metabolites, making their isolation and structural elucidation challenging. Many bioactive compounds are present at very low concentrations, requiring highly sensitive and specific analytical techniques for detection [10], and hundreds of liters of culture. The presence of numerous other metabolites and matrix components can interfere with the isolation process and complicate the identification of target compounds [11].

### 1.2. Cancer and the Search for New Molecules in the Marine Environment

Cancer is a group of diseases characterized by uncontrolled cell proliferation and inhibition of cell death, among other cellular and tissue alterations [12]. Existing treatments have not sufficiently reduced cancer mortality rates, which makes the discovery of new antineoplastic compounds—those that can inhibit tumor growth, kill cancer cells, induce programmed cell death, prevent metastasis, and enhance the immune response—a top priority in cancer research [13].

The National Cancer Institute has reported that only 0.01% of the metabolites isolated from terrestrial sources show cytotoxic effects against cancer cells. In contrast, a hundred more times, approximately 1% of metabolites from aquatic sources exhibit similar activity [14]. This discrepancy can be explained by the exceptional biological and chemical diversity in marine environments, characterized by extreme and unique conditions such as varying pH, intense sunlight, temperature fluctuations, salinity, and the presence of diverse predatory species [14,15]. These harsh conditions contribute to the production of secondary metabolites with distinctive and intricate chemical structures in marine micro- and macro-organisms. These metabolites are often highly oxygenated and unsaturated and may include halogen atoms—uncommon features in terrestrial natural products [16].

In other words, the marine environment’s unique and demanding conditions drive the development of a broader range of complex and diverse chemical compounds with potential antineoplastic properties. These features make marine sources a rich and valuable area for discovering new compounds that may be more effective against cancer than those derived from terrestrial sources [17].

In this study, a methodological strategy of multi-step extraction, column chromatography, gas chromatography coupled to mass spectrometry (GC-MS), high-performance liquid chromatography (HPLC) coupled to a quadrupole time-of-flight mass spectrometry (QTOF-MS), and nuclear magnetic resonance spectroscopy (NMR) analytical techniques were proposed for the identification of compounds with potential antineoplastic activity from *C. malayensis*. The fractionation was bio-directed, and cytotoxicity of the extracts, sub-extracts, and fractions was evaluated in breast, prostate, and lung cancer cell lines, representing the tumor types with the highest incidence and mortality rates worldwide. Finally, the induction of cell death by the most active sub-extract and fractions was analyzed in the most sensitive cells, PC-3, and H1299.

## 2. Results

### 2.1. Maceration Times and Yields of Extracts

Two maceration times were tested. Between 72 and 120 h of maceration, the latter showed a better total methanolic (MET) extraction yield: 72 h = 21.34%, whereas 120 h = 30.67% yield (Table 1). The MET extract was further partitioned into dichloromethane (DCM) and aqueous (AQU) phases. The DCM phase yielded 177 mg after 72 h maceration, and 100 mg after 120 h, suggesting that less non-polar compounds were present in the MET extract over time. Conversely, the AQU phase increased from 170 mg after 72 h to 239 mg after 120 h, indicating a higher retention of polar compounds as maceration progressed.

Hydroalcoholic solution (HYD secondary extract) was used to extract the remaining compounds from the biomass pellet after MET extraction, showing an increase from 32 mg (0.87%) at 72 h to 96 mg (3.41%) at 120 h.

**Table 1:** Yield of the extracts testing different maceration times.

Maceration	Extract/Phase	Weight (mg)	Yield (%)	Origin
72 h 3677 mg dry biomass	MET	785	21.35	Biomass
	DCM	177	-	MET
	AQU	170	-	MET
	HYD	32	0.87	Biomass (extracted pellet)
120 h 2817 mg dry biomass	MET	865	30.72	Biomass
	DCM	100	-	MET
	AQU	239	-	MET
	HYD	96	3.41	Biomass (Pellet)

MET = methanolic extract; HYD = hydroalcoholic secondary extract; DCM = dichloromethane phase (non-polar); AQU = aqueous phase (polar).

### 2.2. Effect of Crude Extract (MET); Secondary Extract (HYD) and Phases (DCM, AQU) on Cell Viability

The methanolic (MET) extract, hydroalcoholic (HYD) secondary extract, and dichloromethane (DCM) and aqueous (AQU) phases were evaluated at a concentration of 100 µg mL^−1^ on four cancer cell lines [prostate (PC-3), cervical (HeLa), breast (MCF-7), and lung (H1299)] and a non-cancerous cell control [human keratinocyte (HaCaT)]. A 0.2% dimethyl sulfoxide (DMSO) solution was used as the vehicle for solubilization and as a negative control. The positive control was paclitaxel (PTX) at 30 nM.

**Figure 1:**
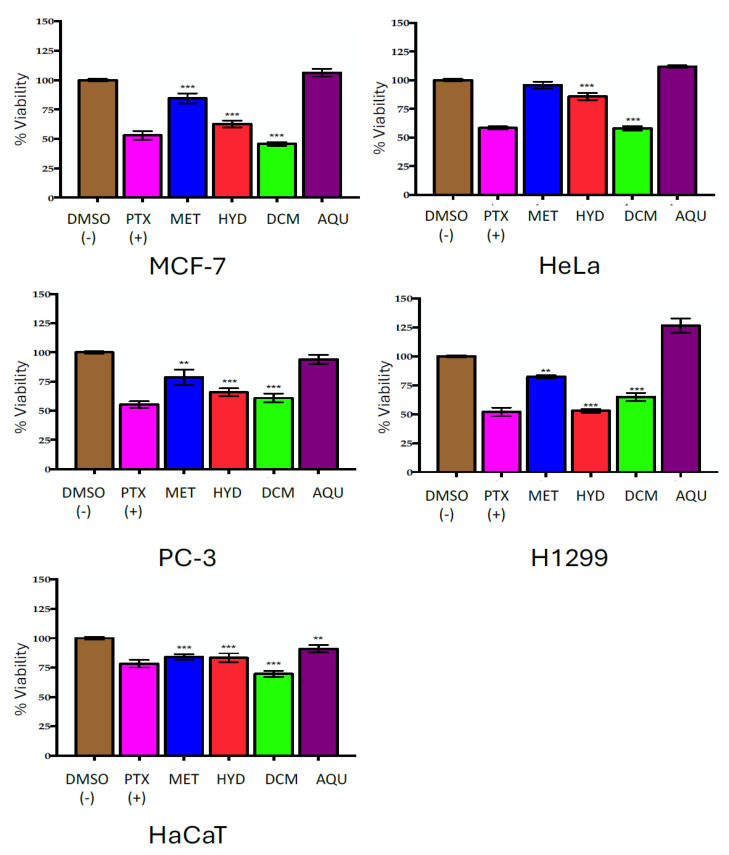
Percentage of cell viability with 100 μg mL^−1^ methanolic MET extract (blue); hydroalcoholic HYD secondary extract (red); dichloromethane DCM (green) and aqueous AQU (purple) phases from *C. malayensis.* Each group represents the mean ± SE; n = 3 vs. DMSO 0.2% (brown), used as negative control and vehicle. Paclitaxel (PTX) 30 nM was used as positive control (pink). Treatments were performed for 72 h. ANOVA, followed by a Dunnett test. Value of *** *p* < 0.0001 ** *p* < 0.005.

The HYD secondary extract and DCM phase exhibited the highest cytotoxic effects, with >35% cell inhibition in all cell lines compared with the control group (DMSO 0.02%, *p* < 0.0001), except in HaCaT, the non-cancerous cell line. Conversely, the MET extract was the least active, showing <25% cell inhibition, while the AQU phase did not have any significant cytotoxic effect in any of the evaluated cell lines (Figure 1, Table 2).

The PC-3 and H1299 cell lines were most sensitive to all crude, secondary extracts and phases, which were used for further analysis.

**Table 2:** Cell inhibition (% ± SD) after 72 h treatments with 100 μg mL^−1^ of methanolic (MET) extract, hydroalcoholic (HYD) secondary extract, and dichloromethane (DCM) and aqueous (AQU) phases from *Coolia malayensis*.

	Cell Line/% Inhibition ± SD				
Treatment	MCF-7	HeLa	PC-3	H1299	HaCaT
**MET**	−15.51 ± 4.15	4.19 ± 3.00	21.40 ± 6.61	17.70 ± 1.23	15.85 ± 2.36
**HYD**	**37.34 ± 2.99**	14.21 ± 3.09	**31.31 ± 3.46**	**47.03 ± 1.61**	16.55 ± 3.80
**DCM**	**54.25 ± 1.59**	**41.92 ± 1.96**	**39.14 ± 3.64**	**35.19 ± 3.48**	**30.40 ± 2.57**
**AQU**	−6.29 ± 3.31	−11.77 ± 1.02	5.93 ± 3.95	−26.50 ± 6.21	9.03 ± 3.26
**PTX (+)**	**46.94 ± 3.64**	**41.68 ± 1.35**	**44.73 ± 2.73**	**45.04 ± 3.73**	21.75 ± 3.22

**In bold:** +30% inhibition. Each group represents the mean ± SE; n = 3 vs. DMSO 0.2%, used as negative control and vehicle. Paclitaxel (PTX) 30 nM was used as positive control. Treatments were performed for 72 h. ANOVA, followed by a Dunnett test.

### 2.3. Effect of Sub-Extracts on Cell Viability

**Figure 2:**
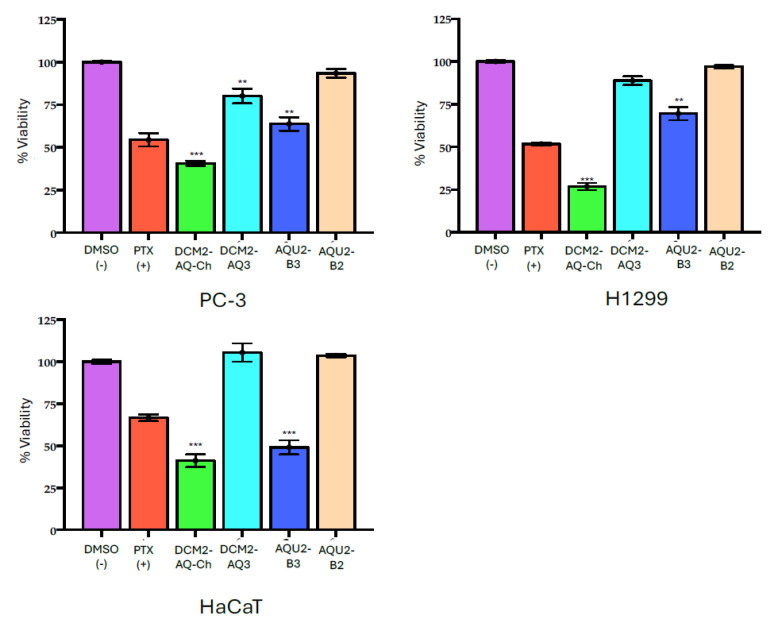
Cell viability of tumor cell lines treated with 50 μg mL^−1^ of sub-extracts DCM2-AQ-Ch (green), DCM2-AQ3 (light blue), AQU2-B3 (dark blue), and AQU2-B2 (light brown), obtained from dichloromethane and aqueous extracts of *C. malayensis*. Each group represents the mean ± SEM; n = 3 vs. DMSO 0.05% (pink), which was used as vehicle and negative control. PTX 30 nM (red) was used as positive control. With a value of *** *p* ˂ 0.0001 ** *p* <0.005 vs. DMSO 0.05% determined by ANOVA, followed by Dunnet test. Treatments were performed for 72 h.

The effect of the sub-extracts DCM2-AQ-Ch, DCM2-AQ3, AQU2-B2, and AQU2-B3 on cell viability (for reference, see Section 4.3) was determined at an exploratory concentration of 50 µg mL^−1^ on PC-3 and H1299 cell lines since those were the most sensitive in the previous test. These fractions were also evaluated on non-cancerous HaCaT cells as the control (Figure 2).

The DCM2-AQ-Ch sub-extract showed the highest activity, with 59.35 ± 2.25% and 73.16 ± 2.35% inhibition on PC-3 and H1299 cell lines, respectively. However, it also exhibited significant effects on HaCaT cells. DCM2-AQ3 sub-extract showed a significant effect on PC-3 cells but not on H1299 and HaCaT cells. Sub-extract AQU2-B3 showed a significant effect on HaCaT cells. Cells treated with AQU2-B2 showed no significant effect. Therefore, the DCM2-AQ-Ch sub-extract, which had the most significant effect on cell viability, was selected for further assays (Table 3).

### 2.4. Chromatographic Fractionation of the Methanolic Crude Extract

Chromatographic fractionation of 200 mg of the MET extract yielded 165 mg in seven fractions (MET fractions), a yield of 82.5% (Table 4).

### 2.5. Effect of MET Fractions on Cell Viability

**Figure 3:**
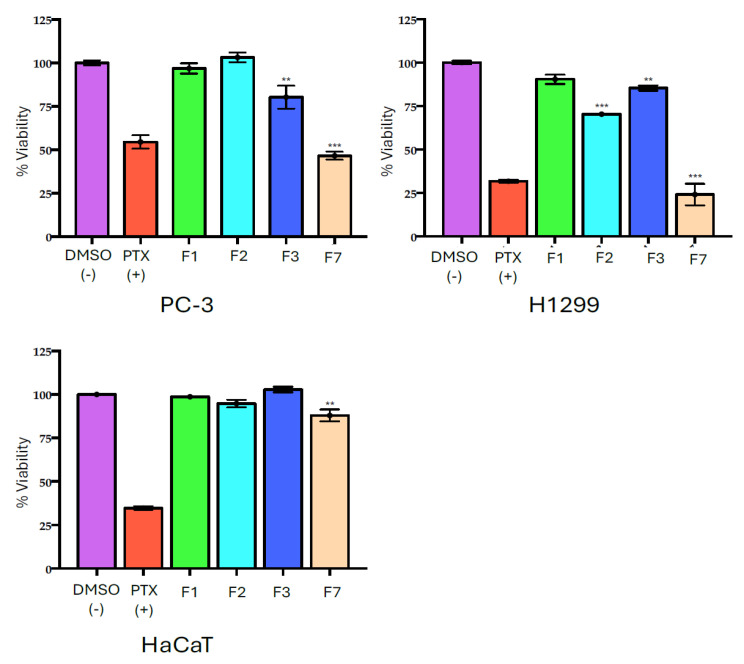
Cell viability of cells treated with 50 μg mL^−1^ fractions from MET extract of *C. malayensis*. F1 (green), F2 (light blue), F3 (dark blue), (F7 (light brown). Each group represents the mean ± SEM; n = 3 vs. DMSO 0.05% (pink) which was used as vehicle and negative control. PTX 30 nM (red) was used as positive control. With a value of *** *p* ˂ 0.0001 ** *p* < 0.005 vs. DMSO 0.05% determined by ANOVA test, followed by Dunnet test. Treatments were performed for 72 h.

The fractions F1, F2, F4, and F7 were evaluated at a concentration of 50 µg mL^−1^ on PC-3 and H1299 cell lines. HaCaT cells were used as the non-cancerous cell control. Fractions F4 and F7 showed the highest cytotoxic effect on PC-3, while H1299 was sensitive only to F7. No fraction affected HaCaT cells (Figure 3 and Table 5), showing an attractive selectivity for cancer cells.

### 2.6. Half-Maximal Inhibitory Concentration of Active Sub-Extract and Fractions

Selectivity indices were calculated to assess the selectivity of the fractions. A high selectivity index indicates that the compound preferentially affects tumor cells compared to non-tumor cells, which is advantageous in developing therapeutic agents.

The IC_50_ values for the most active sub-extract (DCM2-AQ-Ch) and fractions (F4 and F7) were obtained for the H1299 and PC-3 cell lines, as well as the control HaCaT cell line, to calculate their selectivity index. The results in Table 6 indicate that the DCM2-AQ-Ch sub-extract demonstrated the best cytotoxic effect on both cell lines, with IC_50_ values of 17.69 ± 2.38 μg mL^−1^ for H1299 and 21.62 ± 5.73 μg mL^−1^ for PC-3. Fraction F4 exhibited an IC_50_ greater than 100 μg mL^−1^ across all three cell lines, while F7 showed a slightly higher IC_50_ for H1299 (25.16 ± 2.38 μg mL^−1^) and a slightly lower IC_50_ for PC-3 (19.97 ± 1.32 μg mL^−1^) compared to the sub-extract DCM2-AQ-Ch. However, F7 had a better selectivity index (SI), as it had no significant effects on HaCaT at concentrations exceeding 100 μg mL^−1^.

### 2.7. Acridine Orange (AO)-Ethidium Bromide (EB) Double Staining Cell Morphological Analysis

#### 2.7.1. PC-3 Cell Line

**Figure 4:**
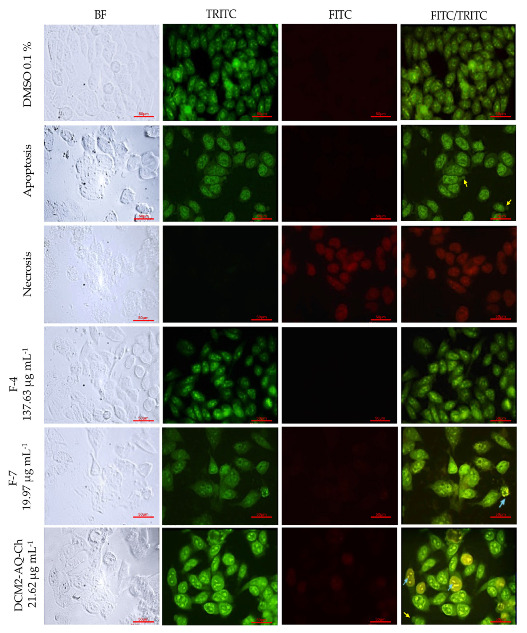
Staining with AO/EB of PC-3 cells to evaluate cell death by fluorescent microscopy (400×). DMSO 0.1% was used as negative control. Apoptosis control cells were treated 30 min with 125 µL H_2_O_2_. Necrosis control cells were treated with water at 95 °C for 10 s. Cells were treated with IC_50_ concentrations of the sub-extract DCM2-AQ-Ch and fractions F4, and F7. Bright green nuclei: viable cells. Yellow arrows indicate the formation of vacuoles; blue arrows show chromatin condensation and DNA fragmentation. BF = bright field; TRITC = tetramethylrhodamine isothiocyanate filter; FITC = fluorescein isothiocyanate filter.

The results of the double AO/EB staining for the PC-3 cell line treated with the IC_50_ concentrations of DCM2-AQ-Ch sub-extract and fractions F4 and F7 are presented in Figure 4. The number of viable cells decreased following DCM2-AQ-Ch sub-extract treatments, which induced morphological changes: the cells were stained green and displayed bright green dots in the nuclei due to chromatin condensation and nuclear fragmentation, resembling the apoptosis control. The formation of vesicles in the plasma membrane of some cells was also observed. These morphological changes are characteristic of cell death by apoptosis; however, further analyses are required for confirmation. Treatments with F7 displayed bright green dots in the nuclei due to slight chromatin condensation and invaginations in some cells. None of the treatments exhibited characteristics of necrosis.

#### 2.7.2. H1299 Cell Line

**Figure 5:**
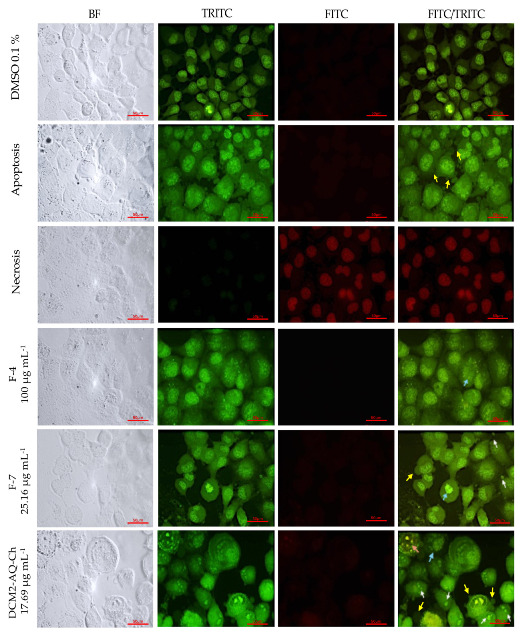
Staining with AO/EB of H1299 cells to evaluate cell death by fluorescent microscopy (400×). DMSO 0.1% was used as negative control. Apoptosis control cells were treated for 30 min with 125 µL of H_2_O_2_. Necrosis control cells were treated with water at 95 °C for 10 s. Cells were treated with the IC_50_ concentrations of the sub-extract DCM2-AQ-Ch and fractions F4 and F7. White arrows indicate apoptotic bodies. Yellow arrows indicate the formation of vesicles. Bright green nuclei: viable cells. Blue arrows show chromatin condensation and DNA fragmentation. BF = bright field; TRITC = tetramethylrhodamine isothiocyanate filter; FITC = fluorescein isothiocyanate filter.

The results of the double AO/EB staining for the H1299 cell line treated with the IC_50_ concentrations of DCM2-AQ-Ch sub-extract and fractions F4 and F7 are shown in Figure 5. Cells treated with the sub-extract DCM2-AQ-Ch exhibited morphological changes, including condensed nuclei, swollen cytoplasm, membrane blebbing, and apoptotic bodies. In contrast, with the control treatment (0.1% DMSO), cells displayed intact nuclear architecture. Fraction F4 also induced morphological changes, evident as bright green dots in the nuclei due to chromatin condensation and the formation of blebs on the plasma membrane of some tumor cells. Cells treated with F7 displayed bright green dots in the nuclei. However, blister formation, similar to the apoptosis control, is not observed.

#### 2.7.3. HaCaT Cell Line

**Figure 6:**
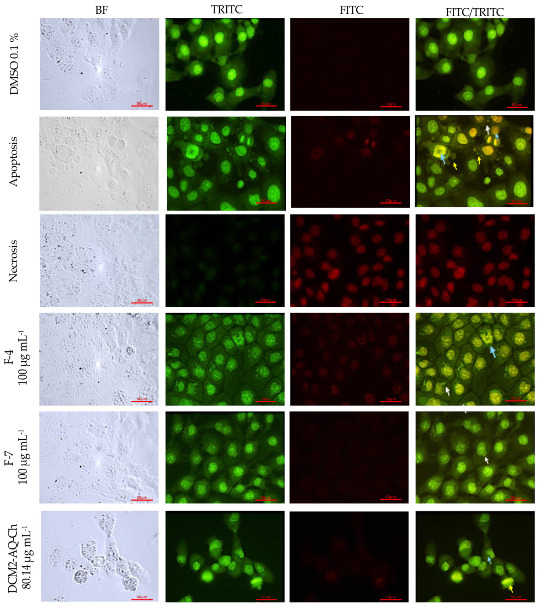
Staining with AO/EB of HaCaT cells to evaluate cell death by fluorescent microscopy (400×). DMSO 0.1% was used as negative control. For apoptosis control, cells were treated for 30 min with 125 µL of H_2_O_2_. Necrosis control cells were treated with water at 95 °C for 10 s. Cells were treated with the IC_50_ concentrations of the sub-extract DCM2-AQ-Ch and 100 µg mL^−1^ of fractions F4 and F7. White arrows indicate apoptotic bodies. Yellow arrows indicate the formation of vesicles. Bright green nuclei: viable cells. Blue arrows show chromatin condensation and DNA fragmentation. BF = bright field; TRITC = tetramethylrhodamine isothiocyanate filter; FITC = fluorescein isothiocyanate filter.

The results of the double AO/EB staining for the HaCaT cell line treated with the IC_50_ concentrations of DCM2-AQ-Ch sub-extract and fractions F4 and F7 are shown in Figure 6. Since the IC_50_ value could not be determined for the fractions, their evaluation was conducted at 100 µg mL^−1^. Cells exhibited morphological anomalies, including chromatin condensation, pyknotic nuclei, and reduced cell density. Although there were no significant changes in cell viability, chromatin condensation was observed in both cases, indicating potential damage. No cells with characteristics of necrosis were observed in any treatment.

### 2.8. Gas Chromatography-Mass Spectrometry (GC-MS) Analysis of the Active Dichloromethane Phase

**Figure 7:**
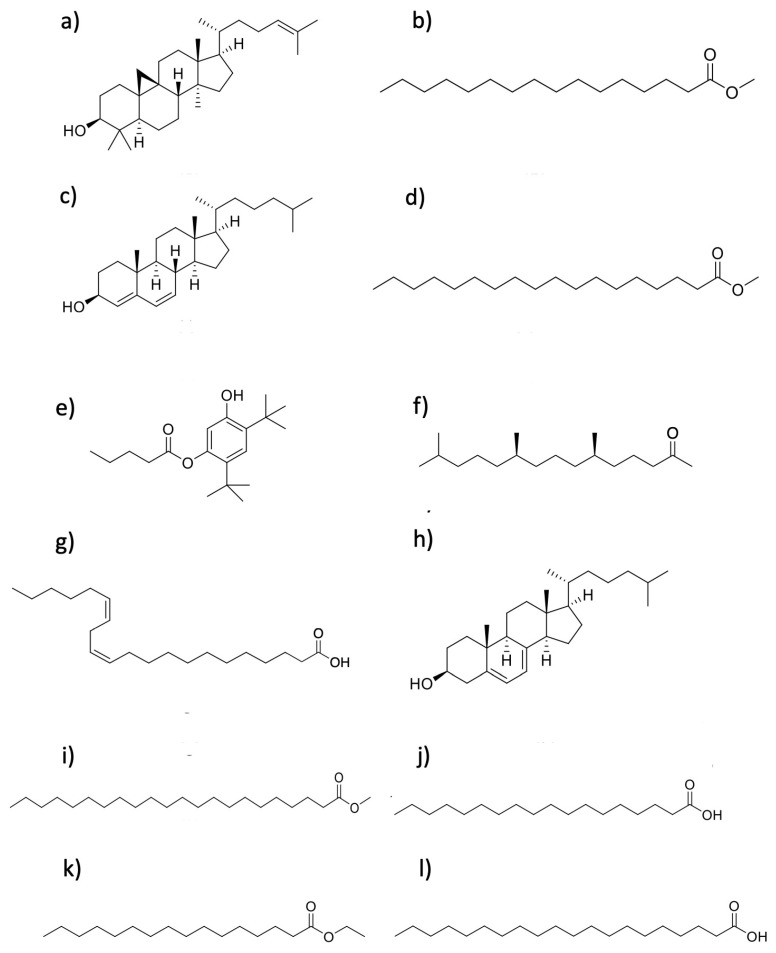
Structures of the GC-MS compounds identified from the active DCM phase. (**a**) Cycloartenol. (**b**) Methyl palmitate. (**c**) (3β)-Cholesta-4,6-dien-3-ol. (**d**) Methyl stearate. (**e**) Pentanoic acid, 5-hydroxy-2,4-di-*t*-butylphenyl ester. (**f**) Phytone. (**g**) *cis*-13,16-Docosadienoic acid. (**h**) (3β)-Cholesta-5,7-dienol. (**i**) Docosanoic acid methyl ester. (**j**) Stearic acid. (**k**) Ethyl palmitate. (**l**) Eicosanoic acid.

A GC-MS analysis was conducted in the most active phase, the dichloromethane-extracted DCM (for reference, see Figure 10 in the Section 4.). According to the retention times and molecular weights and based on the National Institute of Standards and Technology (NIST) database, the most abundant compounds are reported in Table 7. Twelve known bioactive phytochemical compounds were present in the DCM phase of *C. malayensis*. The compounds cycloartenol, methyl palmitate, and (3β)-cholesta-4,6-dien-3-ol were the most abundant. Their structures are presented in Figure 7.

**Table 7:** GC-MS analysis from dichloromethane phase (DCM) of *C. malayensis*.

Figure 7	Compound	MW	RT (Min)	LRI (Calculated)	LRI (Reported)	Relative Abundance %	SI % (Similarity Index)
**a**	**Cycloartenol**	**426.70**	**121.088**	**3487**	**3465** [18]	**24.72**	**99**
**b**	**Methyl palmitate**	**270.50**	**61.233**	**1870**	**1927** [19]	**24.63**	**98**
**c**	**(3β)-Cholesta-4,6-dien-3-ol**	**384.63**	**97.423**	**2742**	**NR**	**15.16**	**95**
d	Methyl stearate	298.50	70.426	2064	2106 [20]	11.51	99
e	Pentanoic acid, 5-hydroxy-2,4-di-*t*-butylphenyl ester	306.45	40.05	1481	1512 [21]	6.91	99
f	Phytone	268.48	57.229	1790	1836 [22]	4.86	98
g	*cis*-13,16-Docosadienoic acid	336.55	87.715	2478	2566 *	3.76	97
h	(3β)-Cholesta-5,7-dienol	384.63	108.088	3067	3160 *	2.62	93
i	Docosanoic acid methyl ester	354.61	89.666	2529	2530 [20]	2.20	95
j	Stearic acid	284.48	74.558	2157	2161 [23]	1.88	92
k	Ethyl palmitate	284.47	64.586	1939	1978 [24]	1.26	98
l	Eicosanoic acid	312.53	81.915	2331	2359 [25]	0.60	96

Data compared with the National Institute of Standards and Technology (NIST) database. MW= Molecular weight; RT = Retention time; LRI = Linear retention index, SI = Similarity Index. * Methyl silicone column, data obtained from NIST. **Bold:** Most abundant compounds.

### 2.9. Nuclear Magnetic Resonance (NMR) Analysis of the Active Extracts and Fractions

#### 2.9.1. Dichloromethane Phase (DCM)

To corroborate the data obtained by GC-MS, the DCM phase was analyzed by ^1^H NMR spectroscopy using deuterated chloroform (CDCl_3_) as the solvent (at 7.26 ppm). The spectrum exhibited signals ranging from 0.88 ppm to 8.11 ppm, corresponding to fatty acid and aromatic protons. Signals between 6.37 ppm and 7.24 ppm displayed characteristics of aromatic protons, while the signal at 8.11 ppm corresponds to a hydroxyl group proton. The observed signals confirmed the presence of aliphatic compounds, but no further information could be obtained from this spectrum since it was a complex sample.

#### 2.9.2. Sub Extract DCM2-AQ-Ch

^1^H and ^13^C NMR (DEPTQ) analyseswere conducted. The spectrum of the sub-extract DCM2-AQ-Ch showed various signals, indicating different types of chemical groups in the complex sample. While some signals correspond to specific groups like methyl and CH_2_ hydrogens, others are less defined, suggesting a mixture of compounds, including sugar residues. Specific signals closely resemble those expected from okadaic acid (OA), hinting at the possible presence of a derivative of this compound in the sub-extract.

#### 2.9.3. Fraction F4

^1^H and ^13^C NMR (DEPTQ) analyses were conducted on fraction F4. This fraction exhibited the highest number of compound groups, as assessed by TLC. The ^1^H NMR spectrum displayed signals ranging from 0.87 ppm to 8.16 ppm. Due to the abundance of signals and the results observed on TLC, we know that fraction F4 contains a too-complex mixture, making it difficult to assign specific signals to individual compounds. The arrangement of signals in the ^1^H spectrum between 3.5 and 4.5 ppm suggests the presence of oxygenated methines and methylenes, while signals in the range of 5.5 to 6.25 ppm may correspond to vinylic protons. Additionally, signals at lower fields, around 7.25 to 8.0 ppm, could correspond to aromatic protons.

#### 2.9.4. Fraction F7

**Figure 8:**
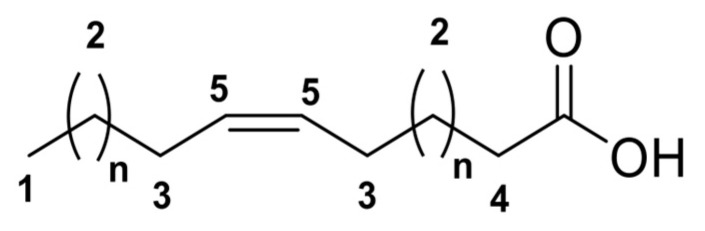
General fatty acid structure.

The ^1^H NMR spectrum of F7) exhibits characteristic signals of fatty acids [21] (Figure 8). Precisely, a signal at 0.88 ppm corresponds to CH_3_ protons (Table 8). Signals within the range of 1.12 to 1.26 ppm can be attributed to CH_2_ protons. The α-protons to the carbonyl group are deshielded, appearing at 2.18 ppm. Additionally, the vinylic protons are downfield as a result of the electron π deshielding effect of the alkene.

### 2.10. HPLC-QTOF-MS Analysis of DCM Phase, DCM2-AQ-Ch Sub-Extract, and Fraction F4

**Figure 9:**
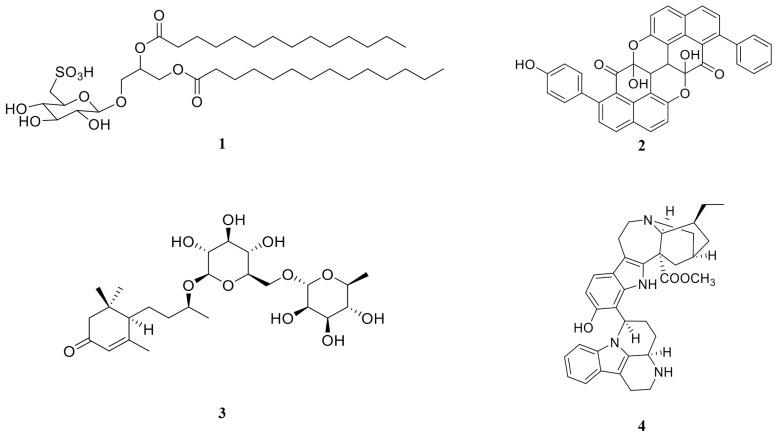

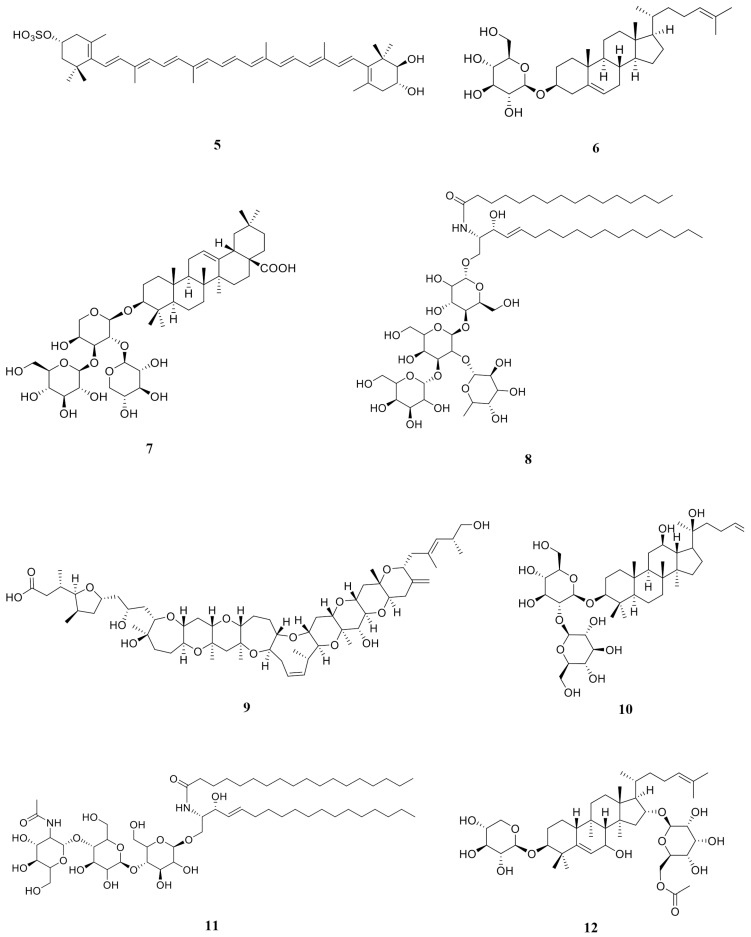

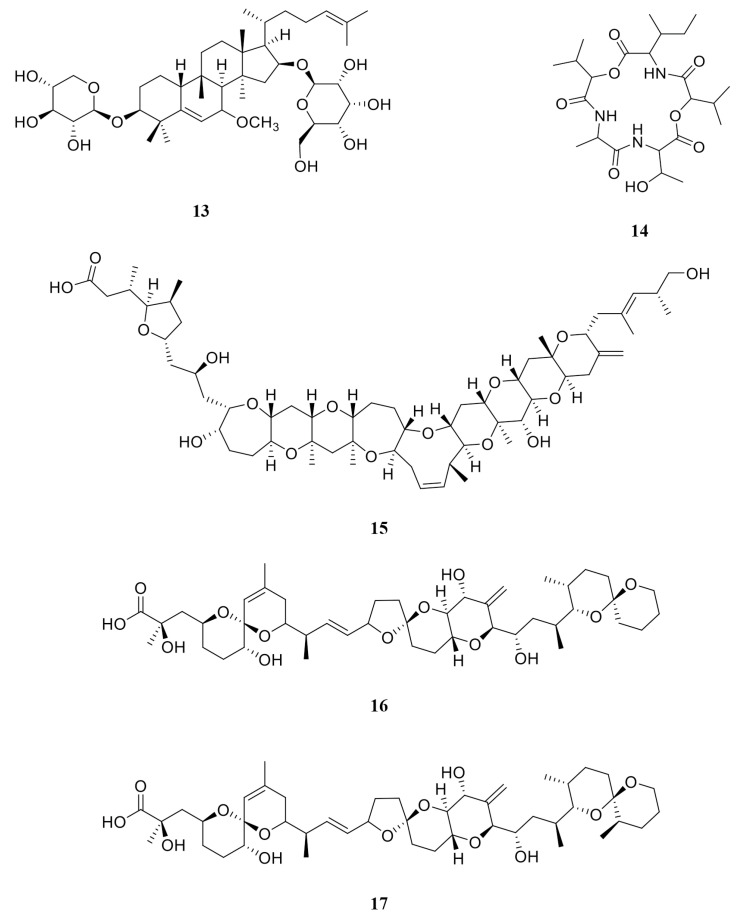
Structures of the identified compounds (>80% probability) by HPLC-QTOF-MS in DCM phase, DCM2-AQ-Ch sub-extract, and fraction F7. For structure names, see Table 9.

More than 200 compounds were recognized (>80% probability) in the DCM phase, DCM2-AQ-Ch sub-extract, and fraction F4 using the combination of Molecular Formula Generation (MFG) and Find by Formula (FBF) function [27]. By selecting the peaks with *m*/*z* ratio tolerance of 5 ppm and a match more significant than 80%, 17 of these compounds were identified (Table 9, Figure 9).

**Table 9:** Main compounds identified (>80% probability) by HPLC-QTOF-MS analysis from the DCM phase, DCM2-AQ-Ch, and fraction F4 from *Coolia malayensis*, according to MassHunter METLIN metabolites-Personal Compound Database and Library (PCDL, Agilent).

Ref. Num. Figure 9	Compound	DCM	DCM2-AQ-Ch	F4	Reported Biological Activity
1	Sulfoquinovosyl diacylglycerol [SQDG]		✓		Anticancer [28,29]
2	4′- Hydroxyanigorootin			✓	Antioxidant and analgesic [30]
3	Blumenol-C-O-[rhamnosyl-(1→6)-glucoside]			✓	Anticancer [31]
4	Bonafousine		✓		Cytotoxic [32]
5	Caloxanthin sulfate		✓		Anticancer, antiviral [33]
6	Stigmasterol glucoside		✓		Anticancer [34]
7	Elatoside E		✓		Cytotoxic [35]
8	Galalpha1–3(Fucalpha1-2)Galbeta1-4Glcbeta-Cer(d18:1/16:0) [globotriaosylceramide]	✓			Inhibitor angiogenesis [36]
9	Gambieric acid B (GAB)	✓			Fungicide [37]
10	Ginsenoside Rg3	✓			Carcinoma [38]
11	GlcNAcbeta1-4Manbeta1-4Glcbeta-Cer(d18:1/18:0) [Lactosylceramide (d18:1/12:0)]	✓			Anticancer [39]
12	Hebevinoside III	✓			Cytotoxic [40]
13	Hebevinoside X	✓			Cytotoxic [41]
14	Sarcodon scabrosus Depsipeptide			✓	Anticancer [42]
15	Gambieric acid A (GAA)	✓			Cytotoxic [43]
16	Okadaic acid (OA)	✓		✓	Anticancer [44]
17	Dinophysistoxin-1 (DTX-1)			✓	Cytotoxic [45]

## 3. Discussion

Benthic dinoflagellates of the genus *Coolia*, particularly the species *C. malayensis*, have been associated with cytotoxic effects and lethal and sublethal impacts on various marine species [46]. Despite these findings, few studies have explored the cytotoxic activity of *C. malayensis* on cancerous cell lines.

As mentioned before, a longer maceration time considerably improved the extraction. Increasing maceration times at room temperature (24 °C) also improved the extraction of compounds [47], so longer maceration times are advised.

### 3.1. Effects of Extracts, Sub-Extracts, and Fractions on Cell Viability

Mitochondrial assays have been used to investigate the effects of crude extracts of *C. malayensis* on cellular function. These assays focus on changes in mitochondrial membrane potential and the cells’ susceptibility to specific processes. Dimethyl sulfoxide extracts of *C. malayensis* can disrupt cellular energy production and increase mitochondrial sensitivity to stimuli, potentially affecting cellular health. Additionally, they have led to a decrease in cell mass [8].

#### 3.1.1. Effect of Crude Extracts on Cell Viability

The cytotoxicity results for the different extracts show varied biological activities across the tested cell lines. Among them, the DCM phase demonstrated the highest inhibition rates against the tested cancer cell lines (MCF-7, HeLa, PC-3, and H1299), highlighting its significant potential as a source of bioactive compounds with anticancer properties. It is important to note that non-polar extracts from dinoflagellates have not been extensively evaluated for their biological activity, even though dichloromethane is an excellent solvent for extracting lipophilic compounds. Additionally, lipophilic compounds that are difficult to eliminate (such as carotenoids and fatty acids) are also extracted, which increases the risk of losing the present lipophilic active compounds. Non-polar phycotoxins, such as yessotoxins, may have some solubility in polar solvents due to the presence of sulfur-ether groups, making these molecules the most polar among lipophilic toxins [48,49]. These features make the separation of active compounds and impurities a challenge.

The HYD secondary extract also demonstrated significant cytotoxicity, particularly against H1299 and PC-3 cell lines, highlighting its potential application in targeting specific cancer types. However, this will require extensive research, separation, and purification processes. This extract contained the most polar metabolites, including carbohydrates, polar lipids, and diacylglycerol derivatives, which are common in marine microorganisms [50,51].

In marine environments, hydrophilic compounds like macrolides, polycyclic ethers, and polyketides, such as those identified in other benthic dinoflagellate genera, such as *Amphidinium* [52], are often associated with *Coolia* spp. and other toxin-producing dinoflagellates [2]. Maitotoxins (MTXs) have been documented [27] among *Coolia* and *Gambierdiscus* species, both benthic dinoflagellates. These toxins are structurally complex, containing multiple functional groups and stereocenters, making them difficult to isolate and characterize. Challenges include limited sample availability, impurities, chemical instability, and degradation [53].

Remarkably, triacylglycerols, despite being less polar, can coexist in polar extracts, and some exhibit potent cytotoxicity. They have been shown to target breast cancer cell lines by stimulating extracellular signal-regulated kinase (ERK) signaling and inducing autophagy in triple-negative breast cancer cells. These molecules also increase dihydroceramide and ceramide levels, inhibiting Akt signaling and selectively inducing cell death in cancer cells. This highlights their potential as therapeutic agents [54].

The MET extract and AQU secondary extract displayed minimal or no cytotoxic activity, even with some negative inhibition percentages (meaning growth stimulation), suggesting that these extracts lack the compounds for significant anticancer effects. The methanolic extract is likely to contain a greater variety of compounds, as it is suitable for extracting both polar compounds and some water-soluble toxins. In this work, the MET extract showed low cytotoxic activity in all the evaluated cell lines. The low cytotoxicity observed could be attributed to the complex metabolic variability, where certain compounds, like antioxidants, carbohydrates, and fatty acids, might exert antagonistic effects, potentially reducing the overall cytotoxicity [55]. However, after 72 h of treatment, the AQU phase showed no significant effect on the tumor cell lines but a very slight effect on HaCaT cells. Phycotoxins with numerous sulfate and hydroxyl groups, such as CTXs and some MTXs, have been identified [56] in such polar extracts; however, no effect was observed in the cell trials.

Intriguingly, the positive control (PTX) consistently inhibited all cell lines, validating the assay’s reliability and serving as a benchmark for the extracts’ activity. Overall, these results emphasize the potential of the DCM and MET as sources of cytotoxic compounds. Therefore, further fractionation is suggested to isolate the active components more effectively.

#### 3.1.2. Effect of Sub-Extracts on Cell Viability

The sub-extract DCM2-AQ-Ch demonstrated the highest inhibition in tumor-derived cell lines PC3 and H1299, with similar inhibition in non-tumorigenic HaCaT cells (Figure 2), indicating potent but non-selective activity. This sub-extract was obtained using a method originally described by McMillan [57] for isolating MTXs and CTXs. Other studies have also successfully used this approach to extract compounds like okadaic acid (OA), a diarrhetic polyether toxin produced by some species of dinoflagellates of the genera *Prorocentrum* (but only by the benthic *Prorocentrum* species, like *P. lima*) and planktonic dinoflagellates from the genus *Dinophysis* [58,59,60]. It is worth mentioning that OA or an OA analog, like dinophysistoxin-1, was identified in the DCM phase and F4 fraction. This is the first report of such phycotoxins being produced by dinoflagellates from the genus *Coolia*. However, more research is needed to ensure this genus can produce them.

McMillan and Bagnis [61] reported that water-soluble and fat-soluble fractions showed the highest toxicity in mice, which was linked to their higher concentrations of MTXs and CTXs [61]. Similarly, in our study, the fat-soluble DCM2-AQ-Ch sub-extract demonstrated the most potent cytotoxic effects, confirming the effectiveness of this method in isolating potent bioactive compounds.

#### 3.1.3. Effect of Methanolic Fractions on Cell Viability

The separation achieved using Sephadex LH-20 with methanol as an isocratic solvent provides valuable insights into the potential composition of bioactive fractions. Given that phycotoxins, which are moderately sized and partially polar, elute in intermediate fractions, and fatty acids, depending on chain length and saturation, can elute in both earlier and later phases, the observed cytotoxic activity in the middle and late fractions of the MET extract suggests the presence of a diverse range of bioactive compounds.

The high cytotoxicity of these fractions against PC-3 and H1299 cell lines indicates that they likely contain a mixture of phycotoxins and fatty acids. Phycotoxins are known for their potent bioactivities, including cytotoxic and anticancer properties [62,63], while fatty acids, particularly long-chain or unsaturated ones, have been associated with modulatory effects on cell membranes and signaling pathways [64]. This combination may contribute to the observed enhanced cytotoxicity.

### 3.2. Acridine Orange (AO)-Ethidium Bromide (EB) Double Staining Cell Morphological Analysis

Morphological anomalies were observed in the PC3 and H1299 tumor-derived cell lines, with HaCaT cells as the control, after treatments with sub-extract DCM2-AQ-Ch and fractions F4 and F7. No signs of necrosis were observed in any of the treatments. The absence of necrosis indicates that the compounds do not cause widespread cytotoxic damage that could affect normal cells or lead to unwanted inflammation [65], which is beneficial for therapeutic purposes.

Significant morphological changes were observed in the tumor-derived cells, such as chromatin condensation and apoptotic body-like structures, suggesting that the treatments induced cell damage through apoptosis-related mechanisms [66]. This type of programmed cell death is preferable in cancer therapies because it eliminates malignant cells while minimizing collateral damage to surrounding tissue [67]. These findings highlight the potential of the compounds in the treatments as therapeutic agents against tumor cells, with a favorable safety profile since they do not induce necrosis in any cells, including normal cells like HaCaT.

### 3.3. Bioactive Compounds Revealed by Gas Chromatography-Mass Spectrometry of the Dichloromethane Phase

Many microalgae species have been attractive sources for producing a wide range of highly valuable products, including polyunsaturated fatty acids (PUFAs), carotenoids, phycobiliproteins, and polysaccharides, which are primary metabolites. In general, microalgae are composed of substantial amounts of lipids (7–23%), proteins (6–71%), and carbohydrates (5–64%). However, these proportions depend on the microalgal species and growth conditions [68].

For these reasons, we analyzed the less polar DCM phase using GC-MS. While the formation of pyrolysis product artifacts is possible, the risk is considered low since the method used is a well-established standard, and the identified compounds have been reported from marine sources. Cycloartenol, methyl palmitate, and (3β)-cholesta-4,6-dien-3-ol as the most abundant compounds (Table 7); these bioactive molecules are likely significant contributors to the observed pharmacological activities of the extract. Sterols, including cycloartenol, are essential for maintaining the stability of cellular lipid bilayers and are significant structural and functional components in marine microorganisms. Cycloartenol (Figure 7a) has shown diverse pharmacological properties, such as anti-inflammatory, antioxidant, antibacterial, and antitumor [69,70]. Its derivatives have demonstrated high cytotoxicity against cancer cell lines like PC-3 and HCT-15, primarily by inhibiting tumor proliferation, inducing apoptosis, and arresting cells in the G2/M phase. However, these derivatives and the DCM phase itself lack selectivity, affecting both cancer and normal cells [71]. Dinoflagellates are also rich in 4-methyl sterols (Figure 7c) [72], known for their broad-spectrum cytotoxic activities against human cancer cell lines [73].

Methyl palmitate (Figure 7b), another prominent compound in the DCM phase, has shown potent cytotoxic effects on tumor cells, such as MCF-7 and HepG-2 [74]. It reduces cell viability by promoting apoptosis and inhibiting metastatic migration by suppressing the NF-κB signaling pathway [75]. This compound, along with cycloartenol, likely contributed to the cytotoxic activity of the DCM phase.

### 3.4. Nuclear Magnetic Resonance Analysis of the Active Extracts and Fractions

To validate the data obtained by GC-MS, the DCM phase was analyzed using ^1^H NMR spectroscopy. Signals in the up-field region confirmed the presence of aliphatic compounds, likely fatty acids. However, the complexity of the sample prevented detailed structural assignments or identification of specific compounds.

The ^1^H and ^13^C NMR (DEPTQ) analyses of the sub-extract DCM2-AQ-Ch revealed a chemically diverse mixture, as evidenced by the variety of signals observed across different spectra regions. Signals corresponding to methyl and CH_2_ groups indicate aliphatic components, while less defined signals suggest a complex mixture that may include sugar residues. These observations suggest that the sub-fraction DCM2-AQ-Ch contains both lipid-derived and sugar-associated molecules, contributing to its chemical diversity.

The analysis of fraction F4 by NMR and thin layer chromatography (TLC) highlighted its noteworthy chemical complexity. Due to overlapping peaks, assigning specific signals to individual compounds is challenging. However, the agreement of specific signals with known patterns for OA and DTX-1 supports their presence in the fraction.

As previously mentioned, the phycotoxins OA and DTX-1 have not been identified in the *Coolia* genus before. These are lipophilic toxins primarily produced by dinoflagellates of the genera *Prorocentrum* (only the benthic species) and *Dinophysis*. Benthic *Prorocentrum* species such as *P. lima* [76], *P. belizeanum* [77], *P. concavum* [78], and *P. hoffmannianum* [79,80] are major producers of OA and DTXs, thriving in sediments and macroalgal habitats. In contrast, planktonic *Dinophysis* species, including *D. acuminata*, *D. fortii*, *D. norvegica*, *D. caudata,* and *D. acuta*, produce OA and DTXs [81,82,83], often causing diarrhetic shellfish poisoning (DSP) in humans [83]. These compounds have shown potential as anticancers due to their ability to inhibit protein phosphatases 1 and 2A, which play key roles in cell signaling pathways. The selective inhibition of subunits that interact with oncoproteins (such as c-Myc or Akt) can destabilize these pathways and promote cell death in cancer cells. Some studies have shown that okadaic acid (OA) induces dose- and time-dependent apoptosis in Jurkat and CCRF-CEM T leukemia cells, characterized by mitochondrial dysfunction, caspase activation, and DNA fragmentation. Additionally, other studies have demonstrated that inhibiting PP2A enhances the effects of anticancer drugs in resistant cells and increases chemotherapy efficacy, particularly in glioblastoma and sarcoma, emphasizing its broader therapeutic potential [84,85,86].

Moreover, both OA and DTX-1 are polyether toxins linked to diarrheic shellfish poisoning (DSP), which poses significant health risks. Besides their role in DSP, these compounds act as neurotoxins, immunotoxins, and tumor promoters. Numerous studies have shown their ability to cause neurotoxicity and immune dysfunction while also encouraging tumor growth in certain cancers [87,88,89]. Despite being potent toxins, ongoing research into their role in cancer and other diseases is uncovering their complexity. Their ability to affect protein phosphatases has led to exciting discoveries about how they can be utilized in medical treatments, creating new opportunities for fighting cancer and other serious diseases [90,91,92].

These compounds, being polyketides with oxygenated and unsaturated functionalities, align well with the observed NMR features. The combined TLC and NMR data highlight the chemically rich nature of fraction F4, making it a potential reservoir of bioactive molecules for further research.

### 3.5. Fatty Acids from F7

Fatty acids (FA) are common in marine microorganisms, but their anticancer effects are not well documented. FA have shown selective toxicity on lung endothelial and epithelial cells, inducing apoptosis or necrosis [93]. This damage is mediated by mechanisms such as signaling through cell surface or nuclear receptors, leading to increased intracellular Ca^++^ concentration [94]. Additionally, these fatty acids can alter membrane structure, transmembrane signaling, and cell cycle regulation [95]. Although marine microorganisms are known to contain fatty acids, their potential as anticancer agents have not been extensively studied.

### 3.6. HPLC-QTOF-MS Analysis of DCM Phase, DCM2-AQ-Ch Sub-Extract, and Fraction F4

The QTOF analysis of DCM, DCM2-AQ-Ch, and F4 revealed more than 200 compounds, identified using the combination of Molecular Formula Generation (MFG) and Find by Formula (FBF) function [27], resulting in the putative identification of 17 specific compounds (>80% probability). These include complex lipids, glycosides, ceramides, carotenoid sulfate, and toxins such as OA and DTX-1, which are known for their toxicity. These compounds include membrane lipids such as [SQDG], lactosylceramide, and globotriaosylceramide, as well as bioactive compounds like gambieric acid A/B.

Gambieric acids (GAs) have previously been reported within the genus *Coolia* and they are recognized for their antifungal potential [96], with very few reports on their cytotoxic effects in human cells. Despite their structural similarity to other cytotoxic phycotoxins, such as YTX, GA-A, and GA-B have not been reported to exhibit toxicity [97,98].

Although YTXs and CTXs have been widely reported within the genus *Coolia*, they were not identified in the three samples analyzed.

While the probability of the compound being present exceeds 80%, compounds like 4′-Hydroxyanigorootin, blumenol-C-O-[rhamnosyl-(1→6)-glucoside], bonafousine, elatoside E, ginsenoside Rg3, and *Sarcodon scabrosus* depsipeptide (2, 3, 4, 7, 10, and 14 in Table 9, respectively), have only been documented in terrestrial plants and fungi [35,99,100,101,102,103]. The isolation of these and/or similar molecules is worth studying since there are huge possibilities of discovering new unreported structures for marine organisms.

The DCM2-AQ-Ch sub-extract exhibited significant activity compared to the DCM phase and fraction F4, likely due to the presence of key metabolites such as (or similar to) bonafousine, caloxanthin sulfate, and stigmasterol glucoside. A partial least squares discriminant analysis (PLS-DA) (Table S18) indicates that these compounds are abundant and crucial for distinguishing between active and inactive fractions, suggesting that they significantly contribute to the observed biological activity [104].

This work highlights the chemical diversity of the extracts and their potential applications in pharmacology, toxicology, and chemical ecology. The detection of toxic compounds like OA and DTX-1 suggests significant biological activity, which is relevant to the study of toxic *Coolia* species. However, although QTOF analysis offers high precision, additional methods, such as using standards or NMR, are necessary to validate the structures and eliminate the possibility of incorrect identifications.

This study confirmed the putative presence of phycotoxins and other characteristic compounds of marine microorganisms. These compounds could confer cytotoxicity on cancer cells and induce morphological changes similar to those observed in apoptotic processes.

## 4. Materials and Methods

### 4.1. Coolia Malayensis Isolation and Biomass Production

*Coolia malayensis* strain CA24 was isolated from a sample of the seagrass *Thalassia testudinum* on October 2017 from Isla Verde in the Veracruz Reef System (19°11′53.66″ N, 96°4′4.23″ W). Morphological identification was conducted through scanning electron microscopy, and molecular identification involved amplification and sequencing of the D1/D2 regions of the large subunit of rDNA [2]. The culture is part of the Chemical Ecology Laboratory Algal Culture Collection (https://www.biodiversidad.gob.mx/fichas-conabio-war/resources/coleccion/1126, accessed on 17 December 2024).

From the established culture, *C. malayensis* cells were transferred to 50, 125, 250, and finally, 1000 mL (with 800 mL culture medium) Erlenmeyer polycarbonate flasks with a 50%-strength GSe medium [105] modified without soil extract. Biomass was produced in static batch cultures at 24 ± 2 °C, with a 12:12 h light/dark cycle and a light intensity of 80 µmol m^−2^ s^−1^. Since dinoflagellates are extremely delicate, no bubbling air was supplied. Harvests were performed every 30 days by centrifugation (Megafuge 40R, Thermo Scientific, Waltham, MA, USA) in 50 mL conical tubes at 3000× *g* and 4 °C for 10 min. The supernatant was discarded, and the biomass was freeze dried (Freezone Benchtop, Labconco, KA, USA) and kept at −20 °C until extraction. A total of 6.48 g of freeze-dried biomass was obtained from 100 L of *C. malayensis* culture (125 flasks, each of 800 mL volume).

### 4.2. Human Cell Cultures and Controls

Prostate (PC-3; ATCC CRL-1435), cervical (HeLa; ATCC^®^ CCL-2), breast (MCF-7; ATCC^®^ HTB-22™), and lung (H1299; ATCC^®^ CRL-5803™) cancer cells were included in this study. Normal human keratinocytes (HaCaT; CLS 300493) were tested as healthy cells controls. Cancer cell cultures were maintained in Dulbecco’s modified essential medium (DMEM; Gibco/Thermo Fisher Scientific, Waltham, MA, USA) with L-glutamine, supplemented with 10% (*v*/*v*) fetal bovine serum (FBS; Gibco, Wellington, New Zealand). Keratinocytes were maintained in a Dulbecco’s Modified Essential Medium/Ham’s Nutrient Mixture (DMEM: F12), supplemented with 10% (*v*/*v*) FBS (Gibco, Wellington, New Zealand). Cultures were kept at 37 °C in a humidified atmosphere of 95% air and 5% carbon dioxide (CO_2_).

Paclitaxel (PTX) (purity ≥95%, HPLC-grade, Sigma Aldrich CAS 33069-62-4, Burlington, MA, USA) was used for the positive controls in all experiments at a concentration of 30 nM. Since all the extracts and fractions were dry (solvent-free), 0.2% and 0.05% DMSO solutions were used as vehicles for crude extracts and fractions, respectively, and as negative controls in all of the experiments.

### 4.3. Extraction and Fractionation

#### 4.3.1. Methanolic Crude Extract

**Figure 10:**
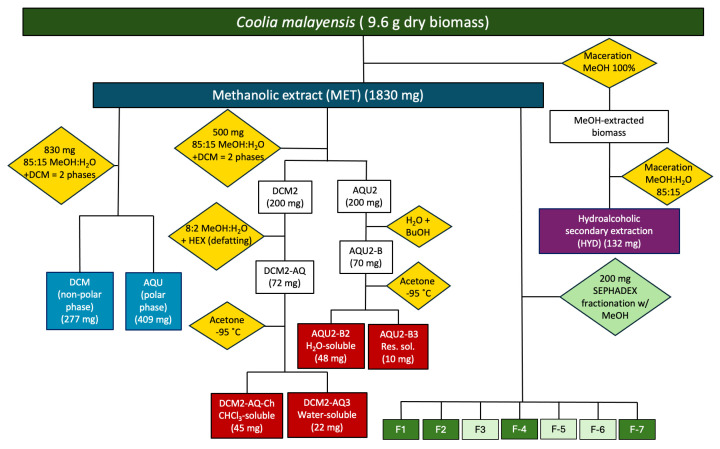
Extraction scheme from freeze-dried *Coolia malayensis* biomass. MeOH: methanol; BuOH: butanol; DCM: dichloromethane; HEX: hexane. Dark blue rectangle: crude extract. Light blue rectangles: polar and non-polar phases. Purple rectangle: secondary extraction. Dark red rectangle: sub-extracts used for in vitro viability cell assays. Yellow diamonds: extraction and phase separation processes. Green diamond: fractionation process. Green rectangles: final fractions from MET crude extract. Dark green rectangles: fractions used for in vitro viability cell assays.

The methanolic extract (MET) was obtained by macerating freeze-dried *C. malayensis* biomass (9.6 g) in pure methanol (99.9% purity, HPLC-grade, Sigma Aldrich, Burlington, MA, USA) for 72 h in the first and 120 h in the second extraction (Figure 10). The mix was sonicated (120 Sonic, Fisher Scientific, Waltham, MA, USA) for 20 min at a 30% amplitude (with a maximum power of 185 W) in continuous mode at 24 °C (room temperature). Then it was vortexed for 1 min, and cell disruption was confirmed by microscopic observations at 100× magnification (Primostar, Zeiss, Jena, Germany). The MET extract was cleared from any possible cellular residues by passing through a chromatographic column with Sephadex LH-20 using HPLC-grade methanol as the mobile phase. Subsequently, it was dried with a rotary evaporator (BÜCHI, Flawil, Switzerland) at 35 °C. Subsequently, the extract was placed in the fume hood for 7 days until dry. Finally, 1830 mg of dry methanol-free MET extract was obtained (Table 1).

#### 4.3.2. Dichloromethane and Aqueous Phases

A portion of the dried methanol-free MET extract (830 mg) was dissolved in 85:15 methanol/distilled-water solution and sonicated (120 Sonic, Fisher Scientific, Waltham, MA, USA) for 20 min at a 30% amplitude (with a maximum power of 185 W) in continuous mode at 24 °C (room temperature). Afterward, HPLC-grade dichloromethane (99.9% purity, Sigma Aldrich, MA, USA) was added in a 1:1 (*v*/*v*) ratio to obtain two phases: the dichloromethane organic phase (DCM) and the aqueous phase (AQU) (Figure 10, Table 1). Both phases were dried using a rotary evaporator (BÜCHI Flawil, Switzerland) at 25 °C (for dichloromethane) and 40 °C (for aqueous). Finally, the phases were placed in the fume hood for 7 days until dry.

#### 4.3.3. Hydroalcoholic Secondary Extract

Since some phycotoxins, such as maitotoxins (MTXs), which have been identified within the genus *Coolia* [27], are highly polar and have been reported from aqueous and hydroalcoholic extracts [106,107], a hydroalcoholic (HYD) secondary extract was obtained. The remaining pellet from the initial methanol extraction was macerated for 72–120 h in a methanol/distilled-water 85:15 solution and sonicated (120 Sonic, Fisher Scientific, Waltham, MA, USA) for 20 min at a 30% amplitude (with a maximum power of 185 W) in continuous mode at 24 °C (room temperature). The biomass was left to precipitate for three days; the supernatant was collected, concentrated, and cleaned, as previously described, to obtain 132 mg of HYD secondary extract (Figure 10, Table 1). Both phases were dried using a rotary evaporator (BÜCHI Flawil, Switzerland) at 25 °C (for dichloromethane) and 40 °C (for aqueous). Finally, the phases were placed in the fume hood for 7 days until dry.

All extracts and phases (MET, HYD, DCM, and AQU) were dried in a fume hood and stored at −18 °C until use.

#### 4.3.4. Sub-Extracts from the Methanolic Crude Extract

Another dichloromethane extraction was performed using 500 mg of the MET extract. It was dissolved in 85:15 methanol/distilled-water solution, and HPLC-grade dichloromethane was added 1:1 (*v*/*v*) to obtain two phases: a second dichloromethane organic phase (DCM2, 200 mg) and a second aqueous phase (AQU2, 200 mg).

The AQU2 phase was dissolved in 15 mL of distilled water and extracted with 15 mL butanol (99.4% purity, J.T Baker Phillipsburg, NJ, USA). The resulting organic phase (AQU2-B, 70 mg) was concentrated, and the aqueous phase of this extraction was discarded.

The phase AQU2-B was dissolved in cold acetone and incubated at −95 °C until a water-soluble precipitate (AQU2-B2, 48 mg) and a residual solution (AQU2-B3, 10 mg) were obtained. Subsequently, the organic phase DCM2 was solubilized in 80:20 methanol/distilled-water solution and extracted with hexane (98.5% purity, Fermont, Monterrey, México) to remove fatty acids (defatting process). The obtained aqueous phase (DCM2-AQ, 72 mg) was evaporated completely, and the hexane phase was discarded. Acetone was added to resuspend the aqueous phase DCM2-AQ and incubated for 24 h at −95 °C. This process was repeated until a chloroform-soluble precipitate (DCM2-AQ-Ch, 45 mg) and acetone-soluble residual solution (DCM2-AQ3, 22 mg) were obtained (Figure 10).

All extracts were evaporated using a rotary evaporator at 30 °C for acetone and 60 °C for butanol extracts and stored at −20 °C for further use. The extracts were monitored by thin-layer chromatography (TLC), using a mobile phase of 7:3 hexane/acetone. Extracts DCM2-AQ-Ch and AQU2-B2 were selected for in vitro cell viability assays since they exhibited fewer bands under ultraviolet light.

#### 4.3.5. Sephadex Fractionation of the MET Extract

MET extract (200 mg) was fractionated in a Sephadex LH-20 chromatographic column (45 × 2.5 cm), and MeOH 100% was the eluent. This process yielded seven fractions (Figure 10). Thin-layer chromatography (TLC) was used to monitor the process and identify collection tubes with similar compositions.

For TLC, 0.25-mm-thick silica gel plates (MERCK; Darmstadt, Germany) were utilized, with combinations of hexane and acetone as mobile phases. Through TLC analysis, collection tubes exhibiting the same chemical compositions were identified and combined.

### 4.4. Chromatographic Fractionation of the Methanolic Crude Extract

Fractionation of the MET extract (200 mg) was performed in a Sephadex LH-20 chromatographic column (45 × 2.5 cm), using MeOH 100% as the eluent [108]. The separation of fractions occurs due to differences in the size of the molecules that elute at different times. Therefore, while the flow of the mobile phase may be isocratic, the goal is to separate the fractions based on the molecules’ sizes. Many marine-derived compounds have been purified through this technique due to the large size of most phycotoxins [109,110,111]. The resin Sephadex LH-20 facilitated the fractionation of the MET extract, which had previously been unsuccessfully fractionated using flash silica 230–400 mesh.

TLC was used to monitor the process and identify collection tubes with similar compositions. The chromatographic separation yielded seven fractions (Figure 10). Fractions F1, F2, F4, and F7 were chosen due to their yield and chromatographic characteristics to determine their effect on cell viability in in vitro assays (Table 4).

### 4.5. In Vitro Cell Viability Assays and Morphological Analysis

#### 4.5.1. Cell Viability Assays

To evaluate cell viability with *C. malayensis* extracts, sub-extracts, and fractions, human cancer cells (5 × 10^3^ cells well^−1^) were seeded in 96-well plates. After 24 h, methanolic (MET) extract, hydroalcoholic (HYD) secondary extract, aqueous (AQU), and dichloromethane (DCM) phases (solvent-free) were evaluated at a concentration of 100 µg mL^−1^. Fractions were evaluated at a concentration of 50 µg mL^−1^.

Active extracts, secondary extracts, sub-extracts, phases, and fractions were evaluated at 50, 25, 12.5, 5.25, and 3.12 µg mL^−1^ for dose–response curves. These were diluted in 0.2 (extract, secondary extract, and phases) and 0.05% (sub-extracts and fractions) DMSO. Therefore, these DMSO concentrations were used as negative controls.

The neutral red uptake assay (Babich and Borenfreund’s method) [112] was used to assess cell viability. Neutral Red Dye (NRD) was prepared at 0.4% (*w*/*v*). After 4 h of incubation with the dye (40 mg mL^−1^) at 37 °C, cells were quickly washed with 100 μL of sterile phosphate buffered saline (PBS) and then with 200 mL of a solution of 50% ethanol, 1% acetic acid, and 49% distilled water. Absorbance was read at 540 nm on a microtiter plate reader.

#### 4.5.2. Half-Maximal Inhibitory Concentration

The half-maximal inhibitory concentration (IC_50_) was determined to assess the inhibitory efficacy of the MET fractions. Non-linear curve fitting functions were applied on normalized dose–response cell viability data obtained from NRU assays; then, IC_50_ values were calculated using GraphPad Prism software version 8.0 (GraphPad Inc., San Diego, CA, USA). The selectivity index (SI) was calculated as the ratio of the IC_50_ for the normal cell line to the IC_50_ for a respective cancerous cell line.

#### 4.5.3. Acridine Orange (AO)-Ethidium Bromide (EB) Double Staining Cell Morphological Analysis

PC3, H1299, and HaCaT cells were seeded at 20 × 10^3^ cells well^−1^ in 24-well plates with glass slides. PC3 and H1299 cells were cultivated in RPMI medium and HaCaT in DMEM F12 medium. They were allowed to adhere overnight at 37 °C in a 5% CO_2_ atmosphere and then were treated for 72 h according to the IC_50_ of the DCM2-AQ-Ch sub-extract and F4 and F7 fractions. The negative control in these trials was 0.1% DMSO, as it was also the vehicle for the sub-extract and the fractions.

For cell controls, the same cell lines were cultured for 71 h in the previously reported media, treated for 30 min with H_2_O_2_ (positive control of apoptosis), or boiled in water at 95 °C for 10 s (positive control of necrosis). Once the 72 h of treatment were reached, all cells (assays and controls) were exposed to a solution of acridine orange (AO) and ethidium bromide (EB) (100 μg mL^−1^ AO, 100 μg mL^−1^ EB), according to reported methods [113]. Cells were observed on a fluorescence microscope (Nikkon Eclipse E 600, Tokyo, Japan) with FITC and TRITC channels and photographed with a digital camera (Canon 600D, Tokyo, Japan).

AO/EB are fluorescent dyes intercalating nucleic acids that, when bound to DNA, give orange (AO) or green (EB) fluorescence. AO can pass through cell membranes, but EB cannot. Necrotic cells stain red but have a nuclear morphology resembling viable cells. Apoptotic cells appear green, and morphological changes, such as the formation of apoptotic bodies, can be observed. The criteria for identification are as follows: viable cells will have a green nucleus with intact structure; early apoptosis cells will exhibit a bright green nucleus showing condensation of chromatin; late apoptosis appears as dense orange areas of chromatin condensation; and orange intact nucleus depicts secondary necrosis.

The experiments were conducted in triplicate in three independent experiments.

### 4.6. Gas Chromatography-Mass Spectrometry Analysis

Gas chromatography (Agilent 6890 Plus, Santa Clara, CA, USA) coupled with a mass spectrometer (Agilent 5379N 30-5550, Santa Clara, CA, USA) (GC-MS) was used for the analysis of the DCM phase. The mass spectrometer operated in electron impact ionization mode at 70 eV and scanned mass-to-charge ratio (*m*/*z*) from 30 to 550. Components were separated using a capillary column (Agilent Technologies HP-5MS, 30 m × 0.25 mm × 0.25 μm). Samples (1 μL) were injected in spitless mode, with a program starting at 50 °C, followed by a 2 °C min^−1^ ramp to 285 °C and a hold time of 20 min. Helium served as the carrier gas at a flow rate of 1 mL min^−1^. Components identification was based on comparing each mass spectrum with those in the N-15598 Mass Spectral Library from the GC-MS instrument and was further corroborated by comparison of the calculated LRI values with those reported in the NIST23 version 3.0 library.

### 4.7. Nuclear Magnetic Resonance Analysis

Nuclear magnetic resonance (NMR) spectra of the DCM phase and its DCM2-AQ-Ch sub-extract were recorded on a Jeol 600 MHz spectrometer using CDCl_3_ as solvent. The NMR spectra of the MET fractions F4 and F7 were recorded on a Bruker 500 MHz spectrometer, using CD_3_OD and CDCl_3_ as solvents, respectively. Chemical shifts were reported in parts per million (ppm) relative to the tetramethylsilane (TMS) signal as an internal standard.

### 4.8. High-Performance Liquid Chromatography-Quadrupole-Time-of-Flight Analysis

High-Performance Liquid Chromatography (Agilent 1200 Infinity, Santa Clara, CA, USA) coupled to a Quadrupole-Time-Of-Flight mass spectrometer analysis (Agilent G6530, Santa Clara, CA, USA) (HPLC-QTOF) was conducted with electrospray ionization (ESI). Samples (10 μL) were injected into a C-8 column (Poroshell 3.0 × 50 mm, 2.7 μM, Agilent, CA, USA) at 30 °C. Gradient elution used water (solvent A) and acetonitrile (solvent B) with 50 mM formic acid at 0.4 mL min^−1^. Elution varied from 5 to 100% solvent B over 10 min; then, for 5 min, 100% solvent B. Initial conditions were restored before each injection. Mass spectrometry conditions: gas temperature of 275 °C and an envelope gas temperature of 300 °C. The capillary voltage was set at 3000 V. Data were acquired in negative ion mode. The mass-to-charge ratio (*m*/*z*) detection range was from 500 to 1500.

Data were processed using Agilent MassHunter Qualitative Analysis 10.0 software. The analysis was performed considering the combination of Molecular Formula Generation (MFG) and Find by Formula (FBF) function [28], utilizing the following criteria: a minimum abundance threshold of 500 counts, a retention time (RT) tolerance of ± 0.15 min, and an *m*/*z* ratio tolerance of ± 10 ppm. The adducts (+CH_3_COO^−^, +HCOO^−^, +Cl^−^, -H^+^) and neutral loss of H_2_O were considered (Tables S1–S3).

Components identification resulted in the putative identification of 17 specific compounds and was based on comparing each *m*/*z* value and each isotopic pattern calculated by the software with those in the library from the HPLC-QTOF instrument. Additionally, these two criteria were compared with those available in the library MassHunter METLIN metabolites-Personal Compound Database and Library (PCDL, Agilent). Peaks with *m*/*z* ratio tolerance of 5 ppm and with a match greater than 80% were selected as probable compounds present in the samples (Tables S1–S3). The compound search formulas were based on Tibiriçá et al. [27] for compounds isolated from *Coolia* species; however, six additional compounds not previously associated with this genus fulfilled the selected criteria. Analysis of the raw data was performed in the free software MetaboAnalyst (https://www.metaboanalyst.ca/home.xhtml, accessed on 20 June 2024).

### 4.9. Statistical Analysis

Cell viability for each assay was calculated with the data obtained from the neutral red uptake (NRU) assay and GraphPad Prism software version 8.0 (GraphPad Inc., San Diego, CA, USA). The experiments were conducted in triplicate in three independent experiments.

Data were processed using analyses of variance (ANOVA) with a 95% confidence level. When the results showed significant differences, the Dunnett test was used to perform further tests.

## 5. Conclusions

An array of compounds with antineoplastic activities were identified from the benthic dinoflagellate *Coolia malayensis*, strain CA24, kept in culture as part of the Chemical Ecology Laboratory Algal Culture Collection. Though complete isolation and full identification of the bioactive molecules were not achieved, this work provides fundamental insight into the chemical composition of this dinoflagellate.

Twelve fatty acid-type compounds and seventeen active metabolites were putatively identified from the DCM phase by GC-MS and HPLC-QTOF-MS analysis. Fraction F7, from the methanolic extract, and the sub-extract DCM2-AQ-Ch exhibited significant cytotoxicity against PC-3 and H1299 cancer cell lines. The sub-extract was the most active and showed some selectivity towards cancer cells. Moreover, these fractions and the sub-extract induced morphological changes similar to those observed in cell death by apoptosis: pyknotic nuclei, membrane blebbing, and swollen cytoplasm. The remarkable activity of the DCM2-AQ-Ch sub-extract can be attributed to the presence of key metabolites, such as bonafousine, caloxanthin sulfate, and stigmasterol glucoside, or molecules with similar structures. These metabolites exhibit cytotoxic and anticancer properties, as reported, which is consistent with our results. In conclusion, *Coolia malayensis* is considered a promising candidate for further studies aimed at the isolation and development of derivatives and bioactive molecules.

The study of chemical compositions from natural sources, often found in small quantities, frequently restricts the isolation of compounds needed for purification and characterization. In these situations, GC-MS and LC-MS analyses serve as effective tools for identifying chemicals by matching their mass and fragmentation patterns with those of known substances and reference standards in databases. However, these techniques have a limitation: they may not conclusively identify new compounds or fully characterize known ones if analysis conditions vary. Despite this drawback, these analytical methods remain the most widely accepted in the scientific community for examining natural sources that are scarce.

## Figures and Tables

**Table 3:** Cell inhibition (% ± SD) after 72 h treatments with 50 μg mL^−1^ of sub-extracts DCM2-AQ-Ch, DCM2-AQ3, AQU2-B3, and AQU2-B2, obtained from dichloromethane and aqueous extracts *Coolia malayensis*.

	Cell Line/% Inhibition ± SD		
Sub-Extract	PC-3	H1299	HaCaT
**DCM2-AQ-Ch**	**59.35 ± 2.25**	**73.16 ± 2.35**	**58.83 ± 3.75**
DCM2-AQ3	19.86 ± 4.38	11.19 ± 2.51	−5.46 ± 3.19
AQU2-B2	6.58 ± 2.59	3.05 ± 0.95	−3.68 ± 0.90
**AQU2-B3**	**36.22 ± 4.27**	**30.48 ± 3.88**	**50.93 ± 4.25**
**PTX (+)**	**54.49 ± 3.84**	**51.76 ± 0.91**	**66.68 ± 1.96**

Each group represents the mean ± SEM; n = 3 vs. DMSO 0.05%. ANOVA, followed by Dunnet test. Treatments were performed for 72 h. **In bold:** +30% inhibition.

**Table 4:** Yield of the MET fractions through Sephadex resin. **In bold**: fractions used for in vitro cell tests.

	200 mg of MET Extract	
Fraction (F)	Weight (mg)	Yield (%)
**F1**	**88**	**44**
**F2**	**20**	**10**
F3	24	12
**F4**	**7**	**3.5**
F5	15	7.5
F6	7	3.5
**F7**	**4**	**2**
**Total yield**	**165**	**82.5**

**Table 5:** Cell inhibition (% ± SD) after 72 h treatments with 50 μg mL^−1^ of MET fractions.

	Cell line/% Inhibition ± SD		
Fraction	PC-3	H1299	HaCaT
F1	3.18 ± 1.92	9.63 ± 4.90	1.12 ± 0.29
F2	−3.05 ± 2.64	29.60 ± 0.11	6.25 ± 2.17
F4	20.73 ± 6.61	14.65 ± 1.55	−2.84 ± 1.56
F7	**53.46 ± 2.28**	**75.88 ± 6.19**	13.05 ± 3.46
PTX	**45.50 ± 3.29**	**68.23 ± 0.88**	**66.32 ± 0.96**

Each group represents the mean ± SEM; n = 3 vs. DMSO 0.05%. ANOVA, followed by Dunnet test. Treatments were performed for 72 h. **In bold:** +30% inhibition.

**Table 6:** IC_50_ values and selectivity index (SI) of the sub-extract DCM2-AQ-Ch and fractions F4 and F7 from *C. malayensis*.

	IC_50_ ± SD (μg mL^−1^)				SI Value			
Cell Line	DCM2-AQ-Ch	F4	F7	PTX	DCM2-AQ-Ch	F4	F7	PTX
H1299	17.69 ± 2.38	>100	25.16 ± 2.38	1.28 × 10^−2^	4.53	-	21.41	2.0
PC-3	21.62 ± 5.73	137.63	19.97 ± 1.32	1.25 × 10^−2^	3.70	0.83	26.96	2.04
HaCaT	80.18 ± 4.90	>100	>100	2.56 × 10^−2^	-	-	-	

Results are expressed as mean ± SD (n = 3).

**Table 8:** ^1^H NMR chemical shifts (δ, ppm) for F7 and reported fatty acids [26].

H	F7	Reported [26]	Peak Asignment
1	0.88	0.88	-CH_3_
2	1.12–1.26	1.20–1.30	-(CH_2_)
3	2.03	2.02	-CH_2_-CH_2_=C
4	2.18	2.29	-CH_2_-C=O
5	5.30	5.30	-HC=CH-

## Data Availability

The original contributions presented in this study are included in the article. Further inquiries can be directed to the corresponding author(s).

## Supplementary Materials

The following supporting information can be downloaded at: https://www.mdpi.com/article/10.3390/md23030127/s1, Table S1: Putative identification of the compounds in the DCM phase of *Coolia malayensis* by HPLC-QTOF-MS; Table S2: Putative identification of the compounds in the DCM2-AQ-Ch sub-extract of *Coolia malayensis* by HPLC-QTOF-MS. Table S3: Putative identification of the compounds in the F4 fraction of *Coolia malayensis* by HPLC-QTOF-MS. Figure S1: HRESIMS data of Sulfoquinovosyl diacylglycerol [SQDG] (**1**); Figure S2: HRESIMS data of 4′-Hydroxyanigorootin (**2**); Figure S3: HRESIMS data of Blumenol-C-O-[rhamnosyl-(1→6)-glucoside] (**3**); Figure S4: HRESIMS data of Bonafousine (**4**); Figure S5: HRESIMS data of Caloxanthin sulfate (**5**); Figure S6: HRESIMS data of Stigmasterol glucoside (**6**); Figure S7: HRESIMS data of Elatoside E (**7**); Figure S8: HRESIMS data of Galalpha1–3(Fucalpha1-2)Galbeta1-4Glcbeta-Cer(d18:1/16:0) [globotriaosylceramide] (**8**); Figure S9: HRESIMS data of Gambieric acid B (**9**); Figure S10. HRESIMS data of Ginsenoside Rg3 (**10**); Figure S11: HRESIMS data of GlcNAcbeta1-4Manbeta1-4Glcbeta-Cer(d18:1/18:0) [Lactosylceramide (d18:1/12:0)] (**11**); Figure S12: HRESIMS data of Hebevinoside III (**12**); Figure S13: HRESIMS data of Hebevinoside X (**13**); Figure S14: HRESIMS data of *Sarcodon scabrosus* depsipeptide (**14**); Figure S15: HRESIMS data of Gambieric acid A (**15**); Figure S16: HRESIMS data of Okadaic acid (**16**); Figure S17: HRESIMS data of Dinophysistoxin-1 (**17**); Figure S18: Metabolic Profiling Across Classes: Heatmap and VIP Scores of Key Compounds from *Coolia malayensis.*
