# Supplementary material for: The Benthic Dinoflagellate Coolia malayensis (Dinophyceae) Produces an Array of Compounds with Antineoplastic Activity in Cells of Tumor Origin
Source: Mar Drugs. 2025 Mar 14;23(3):127. doi: 10.3390/md23030127 (PMC11944075; doi:10.3390/md23030127)
Supplement: Supplementary file 1 [file marinedrugs-23-00127-s001.zip › marinedrugs-3514463-supplementary.pdf]

Supplementary information

The benthic dinoflagellate *Coolia malayensis* (Dinophyceae) Produces an Array of Compounds with Antineoplastic Activity in Cells of Tumor Origin

Itzel B. Morales-Montesinos[1], Maria Yolanda Rios[2], Yordin D. Ocampo Acuña[2], Baldomero Esquivel-Rodríguez[3], Celia Bustos-Brito[3], María del Carmen Osorio-Ramírez[4], Lorena M. Durán-Riveroll[5*], Leticia González-Maya[1*]

[1]Facultad de Farmacia, Universidad Autónoma del Estado de Morelos, Av. Universidad 1001, Col. Chamilpa, Cuernavaca C.P. 62209, México; itzel.moralesm@uaem.edu.mx; letymaya@uaem.mx
[2]Centro de Investigaciones Químicas, IICBA, Universidad Autónoma del Estado de Morelos, Avenida Universidad 1001, Col. Chamilpa, Cuernavaca, Morelos 62209, México; myolanda@uaem.mx; yordin.coampoa@uaem.edu.mx
[3]Instituto de Química, Universidad Nacional Autónoma de México, Circuito Exterior, Ciudad Universitaria, 04510, México; baldo@unam.mx; celia.bustos@iquimica.unam.mx
[4]Departamento de Biotecnología Marina, Centro de Investigación Científica y de Educación Superior de Ensenada, Ensenada 22860, México; osoriorm@cicese.edu.mx
[5]SECIHTI-Departamento de Biotecnología Marina, Centro de Investigación Científica y de Educación Superior de Ensenada, Ensenada 22860, México; lduran@secihti.mx

*Correspondence: lduran@conacyt.mx (L.M.D.-R.); letymaya@uaem.mx (L.G.-M.)

Table S1. Putative identification of the compounds in the DCM phase of *Coolia malayensis* by HPLC-QTOF-MS.

No. in Fig. 9	Name	RT	Relative abundance* (%)	MassHunter METLIN metabolites-Personal Compound Database and Library (PCDL, Agilent)						Diff (ppm) **
				Mass (g/mol)	Formula	Adduct	*m/z*	*m/z* calc.	Score	
8	Galalpha1-3(Fucalpha1-2)Galbeta1-4Glcbeta-Cer(d18:1/16:0) [globotriaosylceramide]	10.788	21.02	1170.5	$C_{58}H_{107}NO_{22}$	M- [-H2O]	1151.7126	1151.7185	83.1	5
9	Gambieric acid B (GAB)	10.54	25.05	1070.65	$C_{60}H_{94}O_{16}$	(M+HCOO)-	1115.6515	1115.6524	83.32	1
10	Ginsenoside C	9.596	8.00	784.49	$C_{42}H_{72}O_{13}$	(M+HCOO)- [-H2O]	811.4849	811.4843	99.09	1
11	GlcNAcbeta1-4Manbeta1-4Glcbeta-Cer(d18:1/18:0) [Lactosylceramide (d18:1/12:0)]	12.445	6.27	1093.4	$C_{56}H_{104}N_2O_{18}$	(M-H)-	1091.7213	1091.7211	97.06	0
12	Hebevinoside III	10.275	18.53	794.48	$C_{43}H_{70}O_{13}$	(M-H)-	793.4744	793.473	96.88	2
13	Hebevinoside X	9.347	4.93	766.48	$C_{42}H_{70}O_{12}$	(M+CH3OO)-	811.4849	811.4849	99.44	0
15	Gambieric acid A (GAA)	8.845	2.29	1056.63	$C_{59}H_{92}O_{16}$	(M+CH3COO)-	1115.6515	1115.6524	85.07	1
16	Okadaic acid (OA)	10.142	0.03	804.46	$C_{44}H_{68}O_{13}$	(M+HCOO)-	863.4841	863.4798	91.53	1
1	Others***		13.89							

* Percentage (%) obtained from the total sum of the areas under the curves.
**The formula to calculate the mass error in parts per million (ppm) is:

$$ppm = \left| \frac{(m/z - m/z\ cal)}{m/z} \right| \times 10^6$$

(m/z): The value obtained experimentally from the mass spectrometer
(m/z cal) The value calculated based on the expected molecular formula and isotopic composition.
***214 peaks with different *m/z* totaling 13.89%.

Table S2. Putative identification of the compounds in the DCM2-AQ-Ch sub-extract of *Coolia malayensis* by HPLC-QTOF-MS.

No. in Fig. 9	Name	RT	Relative abundance* (%)	MassHunter METLIN metabolites-Personal Compound Database and Library (PCDL, Agilent)						Diff (ppm) **
				Mass (g/mol)	Formula	Adduct	*m/z*	*m/z* calc.	Score	
1	Sulfoquinovosyl diacylglycerol [SQDG]	9.49	3.43	738.45	$C_{37}H_{70}O_{12}S$	(M+CH3COO)- [-H2O]	779.4627	779.4628	93.84	0
4	Bonafousine	8.516	6.44	564.31	$C_{35}H_{40}N_4O_3$	(M+CH3COO)- [-H2O]	605.3148	605.3133	85.9	2
5	Caloxanthin sulfate	8.68	20.77	664.38	$C_{40}H_{56}O_6S$	(M+HCOO)-	709.3784	709.378	97.51	1
6	Stigmasterol glucoside	10.42	32.33	546.39	$C_{33}H_{58}O_6$	(M+CH3COO)-	605.4081	605.4059	90.58	4
7	Elatoside E	9.924	2.86	882.5	$C_{46}H_{74}O_{16}$	(M-H)- [-H2O]	863.4839	863.4849	85.34	1
1	Others***		34.17							

*Percentage (%) obtained from the total sum of the areas under the curves.

**The formula to calculate the mass error in parts per million (ppm) is:

$$ppm = \left| \frac{(m/z - m/z\ cal)}{m/z} \right| \text{x } 10^6$$

(m/z): The value obtained experimentally from the mass spectrometer

(m/z cal) The value calculated based on the expected molecular formula and isotopic composition.

***187 peaks with different *m/z* totaling 34.17%.

Table S3. Putative identification of the compounds in the F4 fraction of *Coolia malayensis* by HPLC-QTOF-MS.

No. in Fig. 9	Name	RT	Relative abundance* (%)	MassHunter METLIN metabolites-Personal Compound Database and Library (PCDL, Agilent)						Diff (ppm) **
				Mass (g/mol)	Formula	Adduct	*m/z*	*m/z* calc.	Score	
2	4'-Hydroxyanigorootin	1.012	4.454	590.57	$C_{38}H_{22}O_7$	$(M+CH_3COO)-$	649.1504	649.1504	82.67	0
3	Blumenol-C-*O*-[rhamnosyl-(1→6)-glucoside]	5.998	5.050	518.6	$C_{25}H_{42}O_{11}$	(M+Cl)-	553.2406	553.2421	93.19	3
14	Sarcodon scabrosus Depsipeptide	8.267	9.596	485.27	$C_{23}H_{39}N_3O_8$	(M+HCOO)-	530.2722	530.2719	98.29	0
16	Okadaic acid	10.142	0.239	804.46	$C_{44}H_{68}O_{13}$	(M+HCOO)-	863.4841	863.4798	91.53	1
17	Dinophysistoxin-1	11.083	2.390	818.45	$C_{45}H_{70}O_{13}$	(M+Cl)-	853.4509	853.451	89.42	0
	Others ***		78.270							

*Percentage (%) obtained from the total sum of the areas under the curves.

**The formula to calculate the mass error in parts per million (ppm) is:

$ppm = \left| \frac{(m/z\ - m/\ z\ \ cal\ )}{m/z} \right| \text{x}\ 10^6$

(m/z): The value obtained experimentally from the mass spectrometer

(*m/z cal)* The value calculated based on the expected molecular formula and isotopic composition.

** 185 peaks with different *m/z* totaling 78.27%.

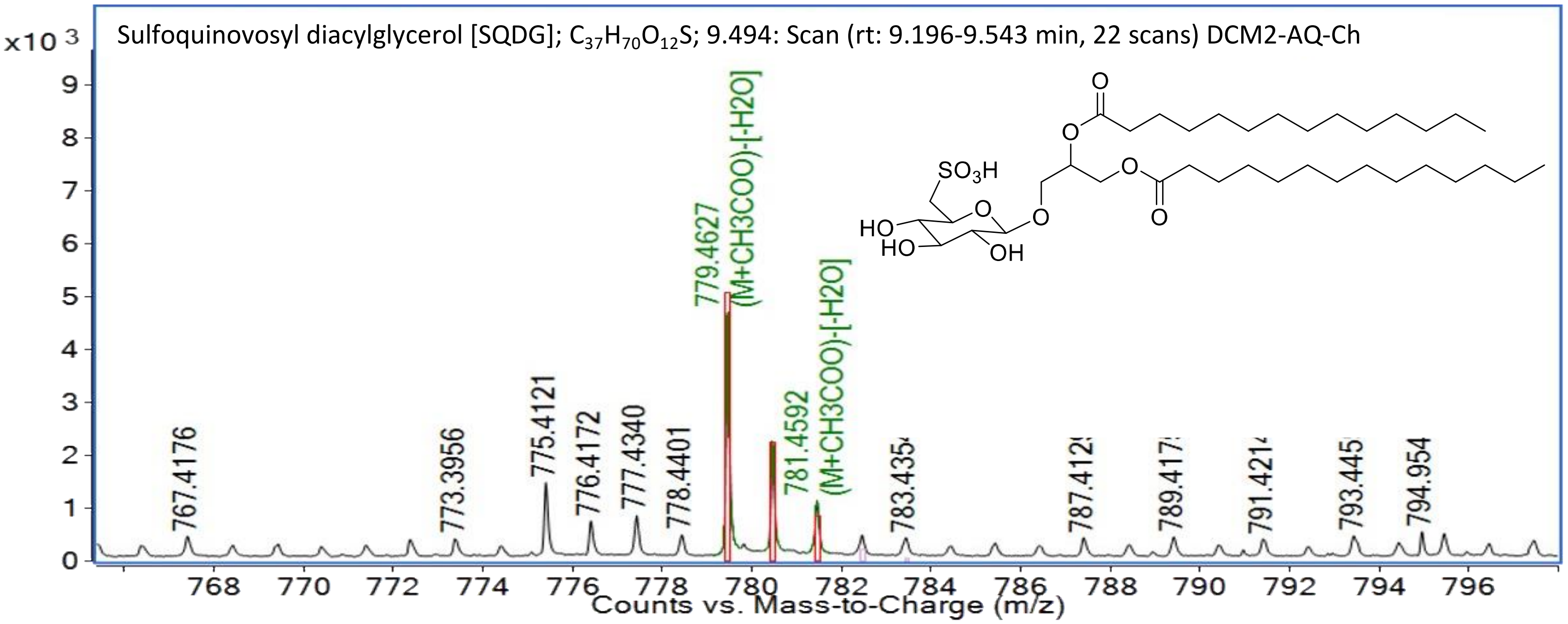

MS spectrum peak list				
m/z	***z***	**Abund**	**Formula**	**Ion**
779.4621	1	28657.68	$C_{37}H_{70}O_{12}S$	$(M+CH_3COO)^-$ [-H_2O]
780.4654	1	10833.71	$C_{37}H_{70}O_{12}S$	$(M+CH_3COO)^-$ [-H_2O]
781.4655	1	3696.69	$C_{37}H_{70}O_{12}S$	$(M+CH_3COO)^-$ [-H_2O]

Predicted isotope match table

Isotope	*m/z*	Calc *m/z*	Diff (ppm)	Calc Abund	Abund %	Calc abund %	Abund sum %	Cal abund sum %
1	779.4621	779.4621	0.0006	5080.1	100	100	58.27	62.11
2	780.4654	780.4654	0.0001	2249.6	47.59	44.28	27.73	27.5
3	781.4655	781.4655	0.0063	849.3	24.03	16.72	14	10.38

Figure S1. HRESIMS data of Sulfoquinovosyl diacylglycerol [SQDG] (**1**)

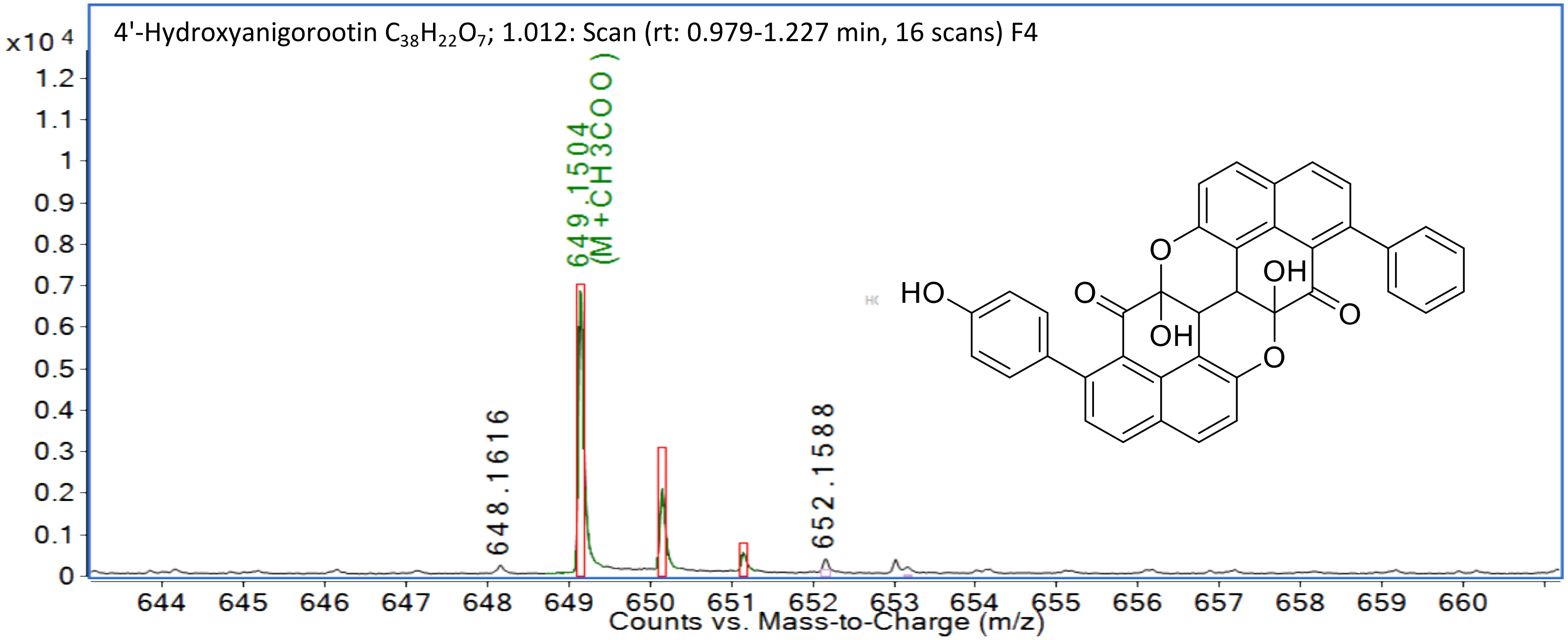

MS spectrum peak list				
m/z	***z***	**Abund**	**Formula**	**Ion**
649.1504	1	790851.8	$C_{38}H_{22}O_7$	(M+CH_3COO)-
650.1538	1	260564.2	$C_{38}H_{22}O_7$	(M+CH_3COO)-
651.1568	1	58496.8	$C_{38}H_{22}O_7$	(M+CH_3COO)-

Predicted isotope match table

Isotope	*m/z*	Calc *m/z*	Diff (ppm)	Calc Abund	Abund %	Calc abund %	Abund sum %	Cal abund sum %
1	649.1504	649.1504	0	6278.8	100	100	72.3	64.46
2	650.1531	650.1538	0.0007	2756	30.02	43.89	21.71	28.29
3	651.1522	651.1568	0.0046	706.3	8.28	11.25	5.99	7.25

Figure S2. HRESIMS data of 4'-Hydroxyanigorootin (**2**)

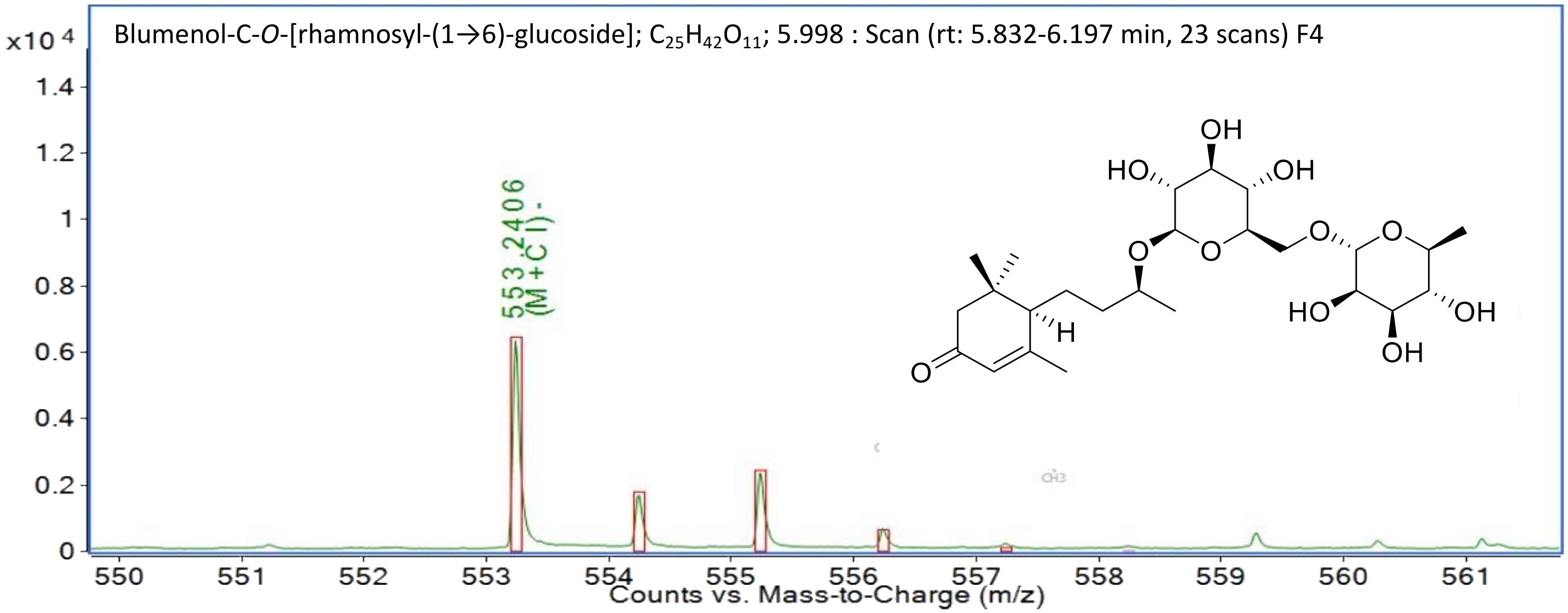

MS spectrum peak list				
m/z	***z***	**Abund**	**Formula**	**Ion**
553.2406	1	6397.4	$C_{25}H_{42}O_{11}$	(M+Cl)
555.2387	1	2401.1	$C_{25}H_{42}O_{11}$	(M+Cl)
554.2438	1	1727.8	$C_{25}H_{42}O_{11}$	(M+Cl)
556.2414	1	702.4	$C_{25}H_{42}O_{11}$	(M+Cl)
557.2372	1	253.2	$C_{25}H_{42}O_{11}$	(M+Cl)

Predicted isotope match table								
**Isotope**	***m/z***	**Calc *m/z***	**Diff (ppm)**	**Calc Abund**	**Abund %**	**Calc abund %**	**Abund sum %**	**Cal abund sum %**
1	553.2406	553.2421	0.0015	6454	100	100	55.72	56.21
2	555.2387	555.2406	0.0019	2453.4	37.53	38.01	20.91	21.37
3	554.2438	554.2455	0.0017	1803.3	27.01	27.94	15.05	15.71
4	556.2414	556.2434	0.002	638.6	10.98	9.89	6.12	5.56
5	557.2372	557.2455	0.0083	132.5	3.96	2.05	2.2	1.15

Figure S3. HRESIMS data of Blumenol-C-O-[rhamnosyl-(1→6)-glucoside] (**3**)

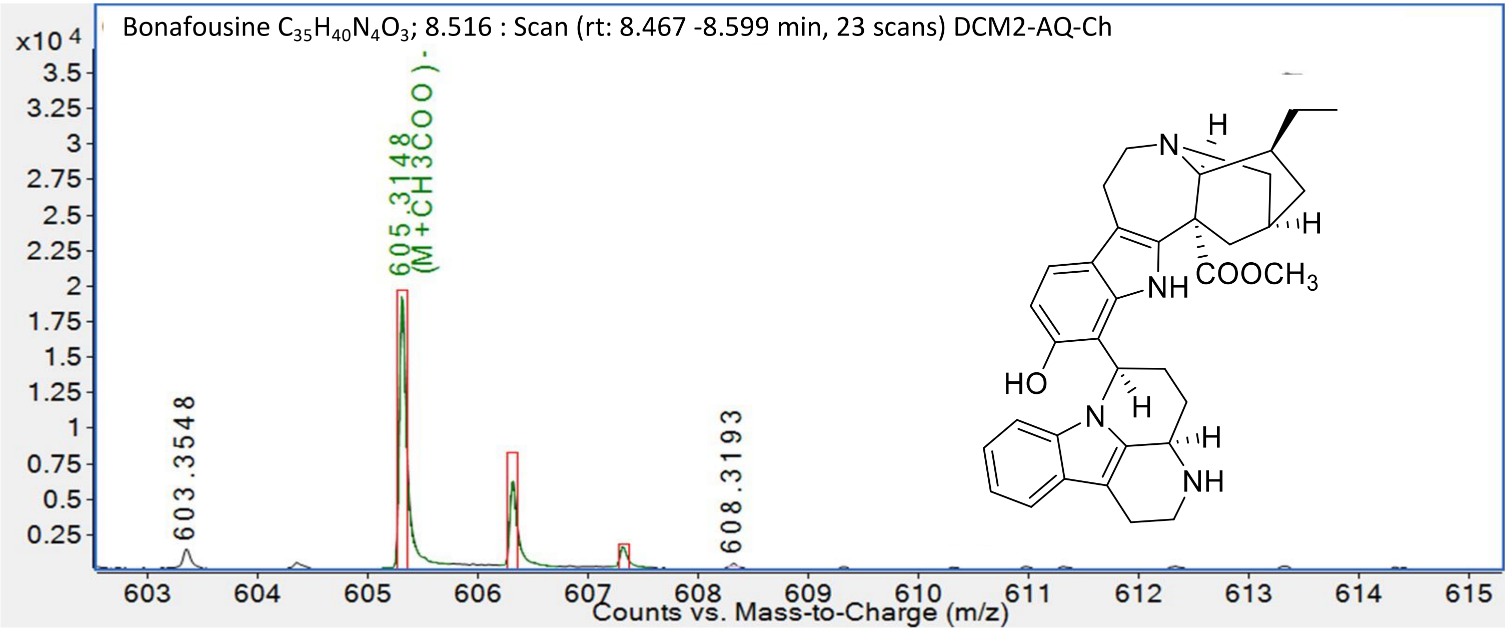

MS spectrum peak list				
m/z	***z***	**Abund**	**Formula**	**Ion**
605.3148	1	19696.3	$C_{35}H_{40}N_4O_3$	(M+CH_3COO)-[-H_2O]
606.3185	1	6338.5	$C_{35}H_{40}N_4O_3$	(M+CH_3COO)-[-H_2O]
607.3184	1	1679.9	$C_{35}H_{40}N_4O_3$	(M+CH_3COO)-[-H_2O]

Predicted isotope match table

**Isotope**	***m/z***	**Calc *m/z***	**Diff (ppm)**	**Calc Abund**	**Abund %**	**Calc abund %**	**Abund sum %**	**Cal abund sum %**
1	605.3148	605.3133	0.003	22882.8	100	100	64.35	66.51
2	606.3185	606.3165	-0.005	8775.7	42.9	38.35	27.61	25.51
3	607.3184	607.3195	-0.004	2295.4	10.65	10.03	6.86	6.67

Figure S4. HRESIMS data of Bonafousine (**4**)

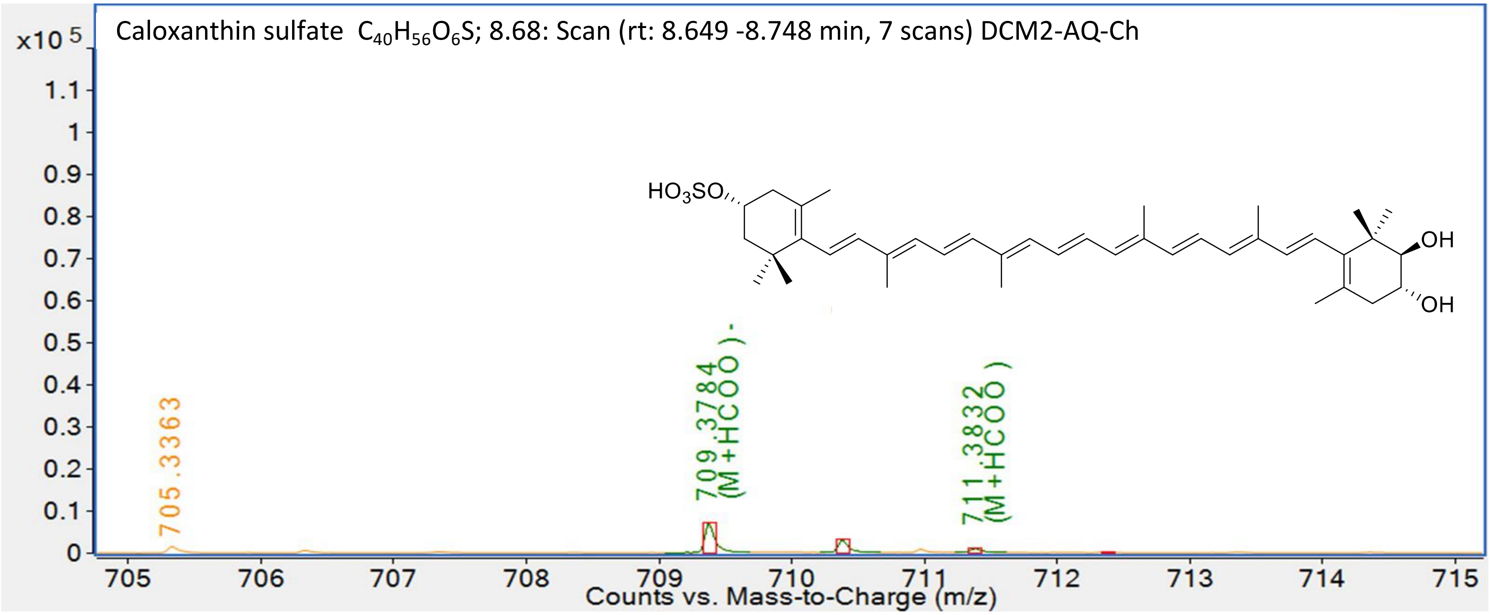

MS spectrum peak list				
m/z	*z*	Abund	Formula	Ion
709.3784	1	7190.1	$C_{40}H_{56}O_6S$	(M+HCOO)-
712.3688	1	340.4	$C_{40}H_{56}O_6S$	(M+HCOO)-
710.3832	1	3134.4	$C_{40}H_{56}O_6S$	(M+HCOO)-
711.3832	1	1122.5	$C_{40}H_{56}O_6S$	(M+HCOO)-

Predicted isotope match table								
Isotope	*m/z*	Calc *m/z*	Diff (ppm)	Calc Abund	Abund %	Calc abund %	Abund sum %	Cal abund sum %
1	7190.1	709.378	0.0004	7062.6	100	100	61	59.92
2	340.4	712.3825	-0.0137	304.1	4.73	4.31	2.89	2.58
3	3134.4	710.3813	0.0019	3255.5	43.59	46.09	26.59	27.62
4	1122.5	711.3814	0.0018	1165.2	15.61	16.5	9.52	9.89

Figure S5. HRESIMS data of Caloxanthin sulfate (**5**)

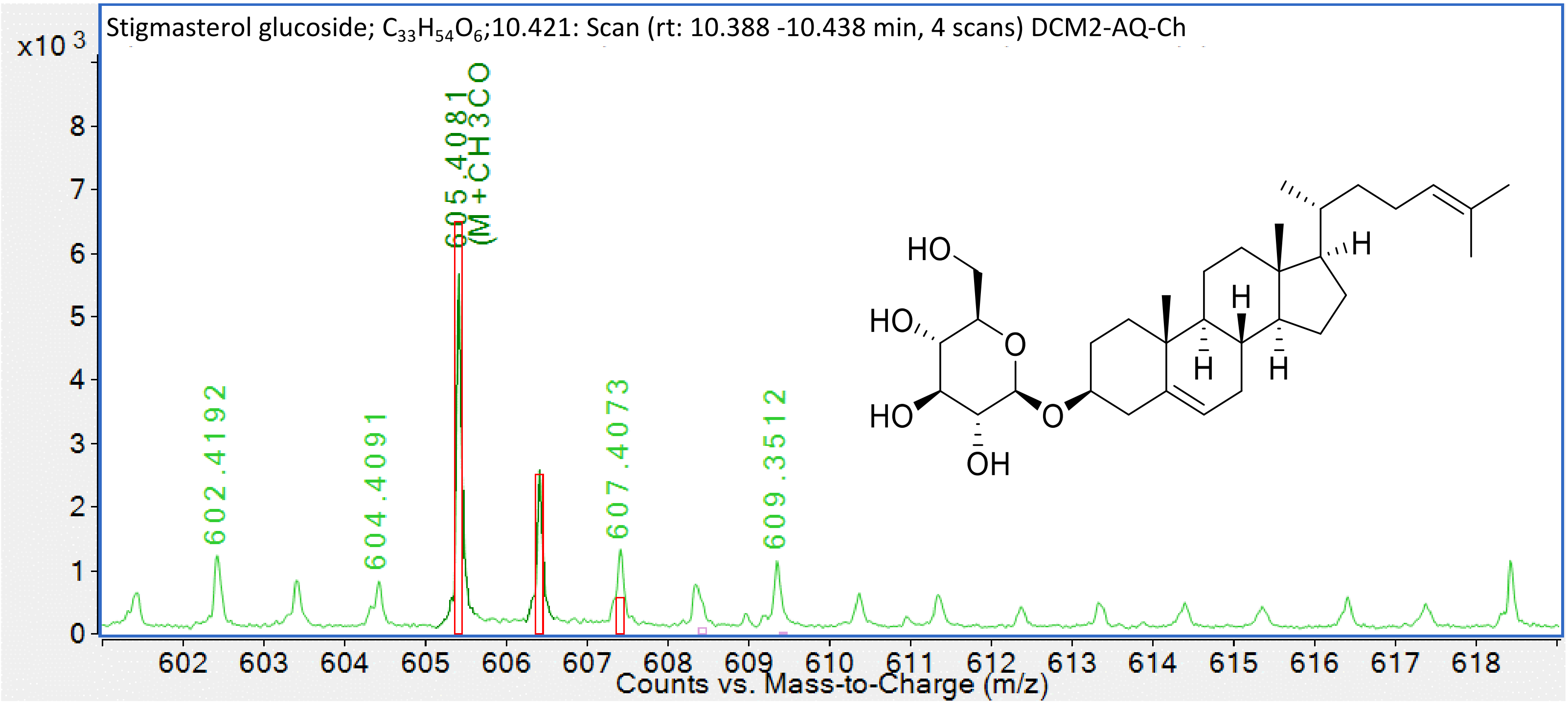

MS spectrum peak list				
m/z	***z***	**Abund**	**Formula**	**Ion**
605.4081	1	5671.4	$C_{33}H_{54}O_6$	$(M+CH_3COO)^-$
606.4105	1	2583.4	$C_{33}H_{54}O_6$	$(M+CH_3COO)^-$
607.4073	1	1336.9	$C_{33}H_{54}O_6$	$(M+CH_3COO)^-$

Predicted isotope match table								
**Isotope**	***m/z***	**Calc *m/z***	**Diff (ppm)**	**Calc Abund**	**Abund %**	**Calc abund %**	**Abund sum %**	**Cal abund sum %**
1	605.4081	605.4059	0.0022	6490.1	100	100	59.13	67.66
2	606.4105	606.4093	0.0012	2519.2	45.55	38.82	26.93	26.26
3	607.4073	607.4122	-0.0049	582.3	23.57	8.97	13.94	6.07

Figure S6. HRESIMS data of Stigmasterol glucoside (**6**)

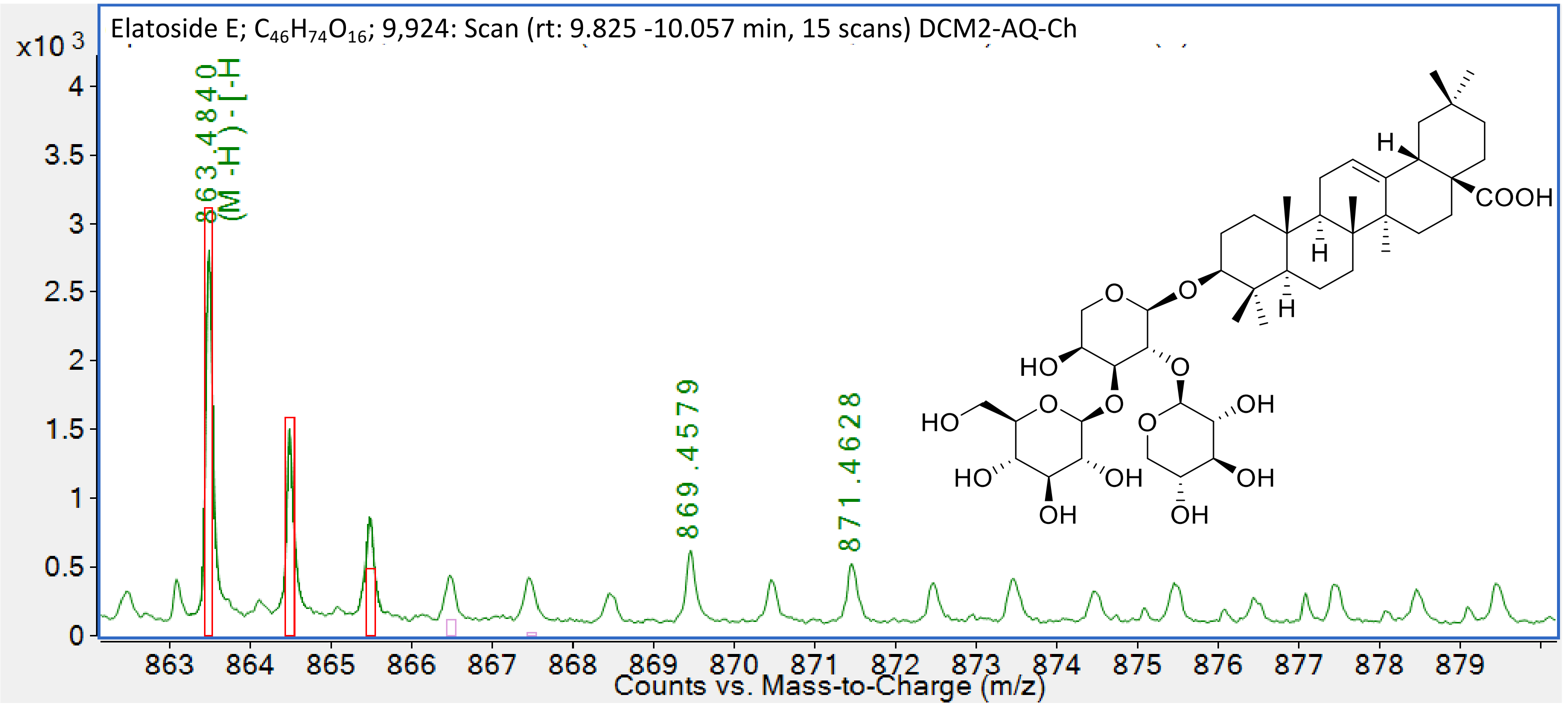

MS spectrum peak list				
m/z	*z*	Abund	Formula	Ion
863.4839	1	2813.5	$C_{46}H_{74}O_{16}$	M-H)-[H_2O]
864.4882	1	1512.5	$C_{46}H_{74}O_{16}$	M-H)-[H_2O]
865.4778	1	870.2	$C_{46}H_{74}O_{16}$	M-H)-[H_2O]

Predicted isotope match table

Isotope	*m/z*	Calc *m/z*	Diff (ppm)	Calc Abund	Abund %	Calc abund %	Abund sum %	Cal abund sum %
1	863.4839	863.4798	0.0041	3110.9	100	100	54.14	59.87
2	864.4882	864.4833	0.0049	1590.9	53.76	51.14	29.11	30.62
3	865.4778	865.4862	-0.0084	494.3	30.93	15.89	16.75	9.51

Figure S7. HRESIMS data of Elatoside E (**7**)

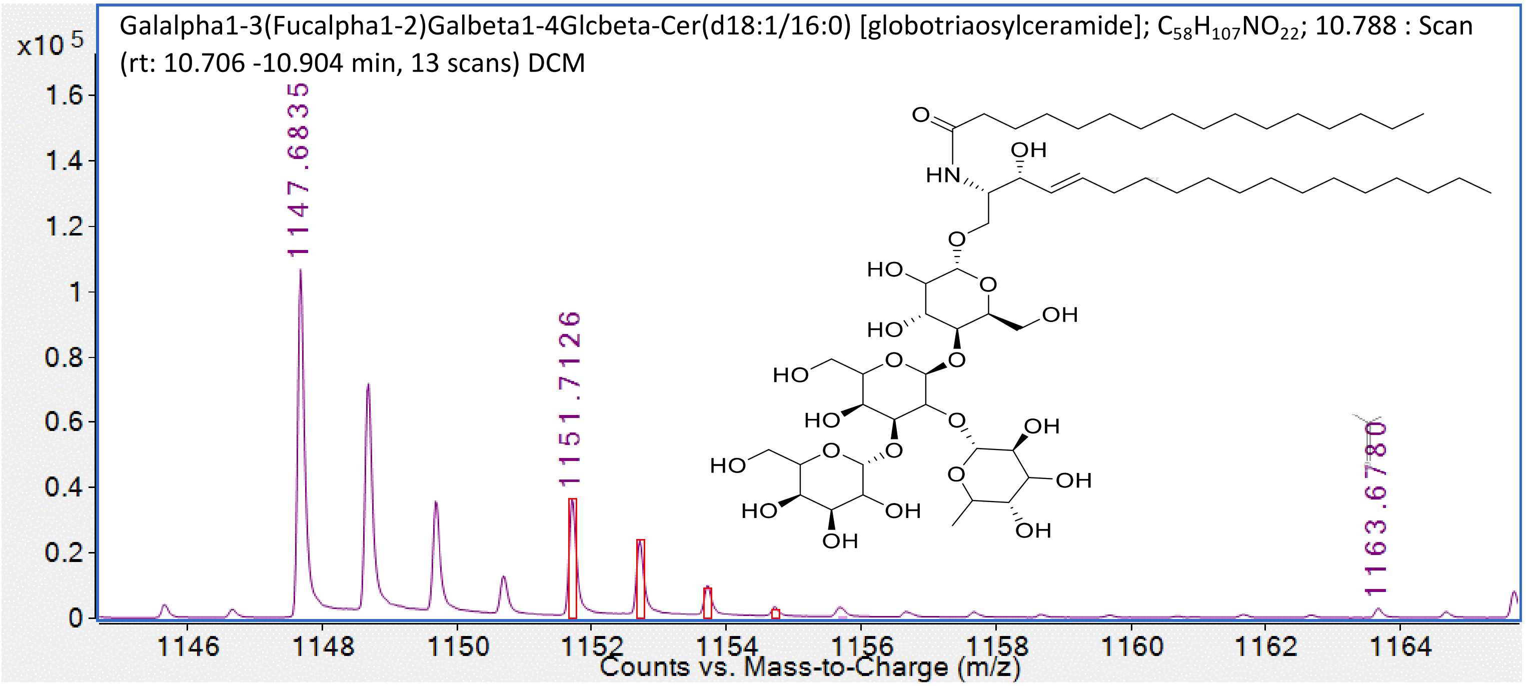

MS spectrum peak list				
m/z	***z***	**Abund**	**Formula**	**Ion**
1151.7126	1	56401.61	$C_{58}H_{107}NO_{22}$	M-[H_2O]
1152.7167	1	14991.78	$C_{58}H_{107}NO_{22}$	M-[H_2O]
1153.7195	1	3122.34	$C_{58}H_{107}NO_{22}$	M-[H_2O]
1154.7217	1	150.88	$C_{58}H_{107}NO_{22}$	M-[H_2O]

Predicted isotope match table

Isotope	*m/z*	Calc *m/z*	Diff (ppm)	Calc Abund	Abund %	Calc abund %	Abund sum %	Cal abund sum %
1	1151.7126	1151.7185	0.001	36623.9	100	100	50.78	50.64
2	1152.7167	1152.7218	0.001	23843.7	64.76	65.1	32.88	32.97
3	1153.7195	1153.7248	0.003	9217.5	24.67	25.17	12.53	12.75
4	1154.7217	1154.7276	0.005	2632.5	7.5	7.19	3.81	3.64

Figure S8. HRESIMS data of Galalpha1-3(Fucalpha1-2)Galbeta1-4Glcbeta-Cer(d18:1/16:0) [globotriaosylceramide] (**8**)

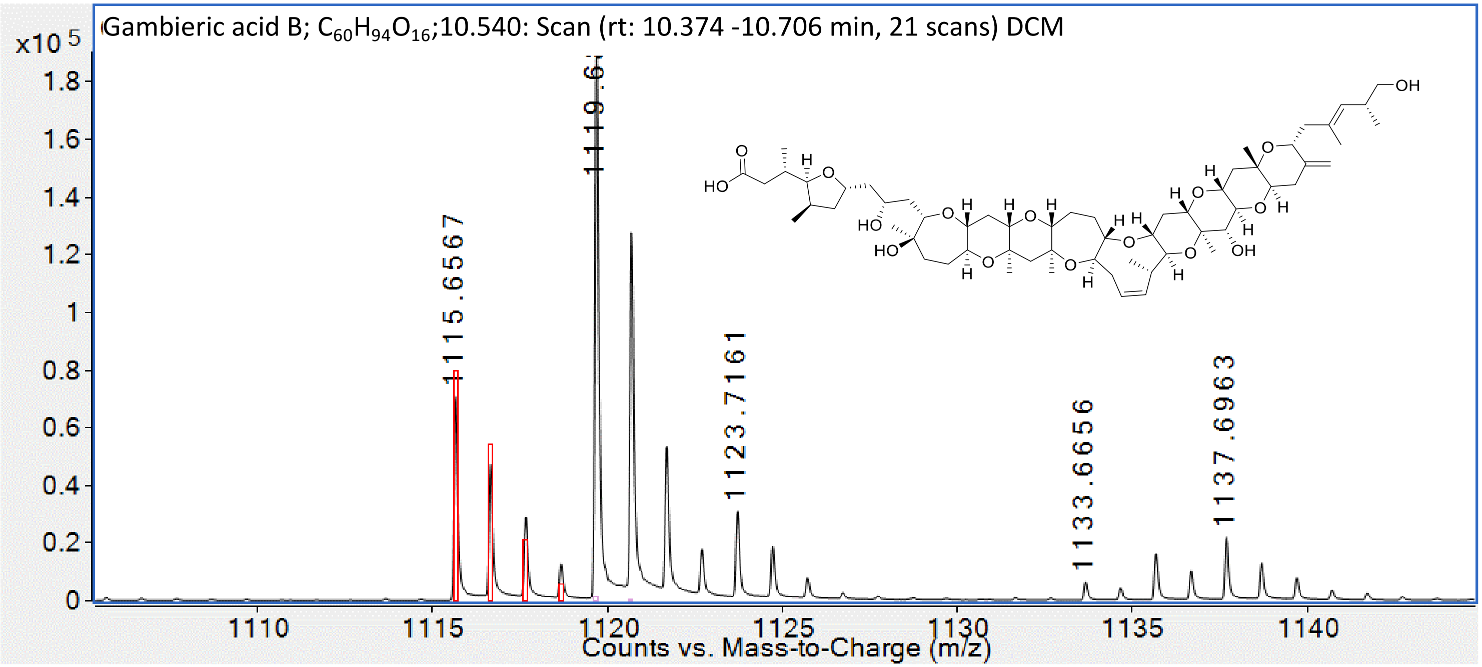

MS spectrum peak list				
m/z	***z***	**Abund**	**Formula**	**Ion**
1115.6567	1	71415.7	$C_{60}H_{94}O_{16}$	(M+HCOO)-
1116.6598	1	47230.9	$C_{60}H_{94}O_{16}$	(M+HCOO)-
1117.6661	1	29420.9	$C_{60}H_{94}O_{16}$	(M+HCOO)-
1118.671	1	12811.4	$C_{60}H_{94}O_{16}$	(M+HCOO)-

Predicted isotope match table								
**Isotope**	***m/z***	**Calc *m/z***	**Diff (ppm)**	**Calc Abund**	**Abund %**	**Calc abund %**	**Abund sum %**	**Cal abund sum %**
1	1115.6567	412.3397	0.0043	79841.1	100	100	44.39	49.63
2	1116.6598	413.3431	0.004	54095.7	66.14	67.75	29.36	33.63
3	1117.6661	414.3459	0.0072	20994.4	41.2	26.3	18.29	13.05
4	1118.671	415.3486	0.0092	5947.7	17.94	7.45	7.96	3.7

Figure S9. HRESIMS data of Gambieric acid B (**9**)

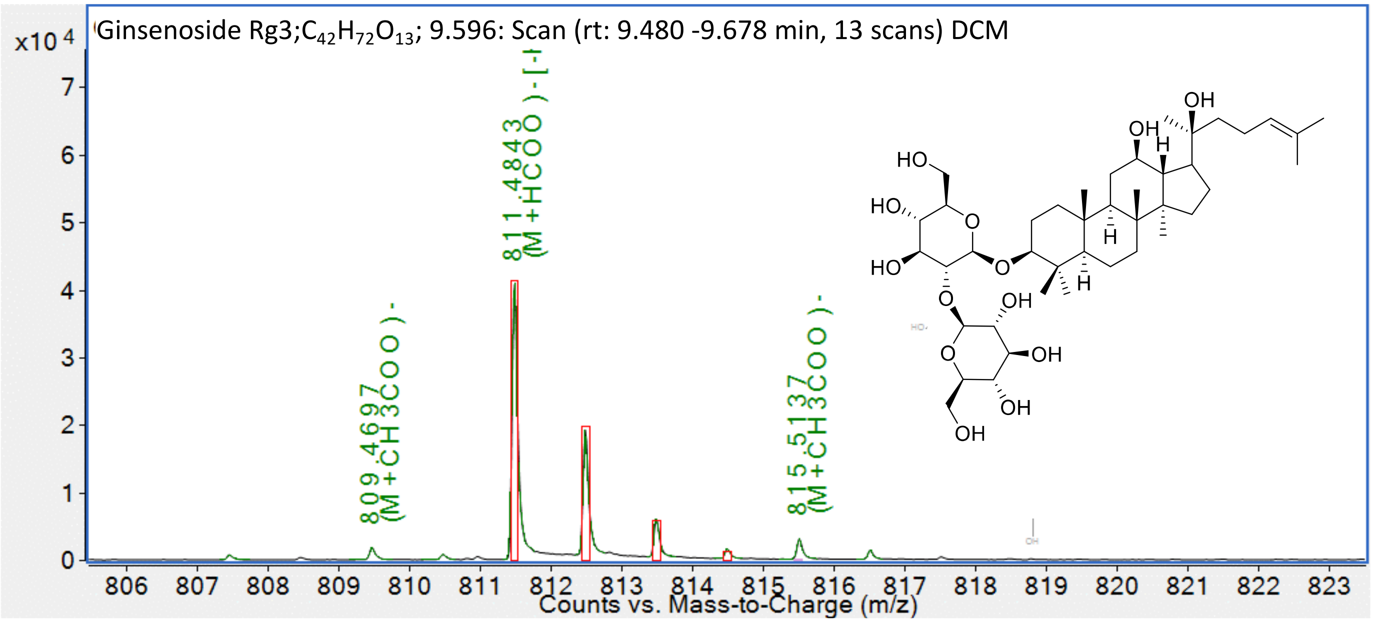

MS spectrum peak list				
m/z	*z*	Abund	Formula	Ion
811.4843	1	41236.9	$C_{42}H_{72}O_{13}$	(M+HCOO)- [-H_2O]
812.4875	1	19374.5	$C_{42}H_{72}O_{13}$	(M+HCOO)- [-H_2O]
813.4905	1	6246.3	$C_{42}H_{72}O_{13}$	(M+HCOO)- [-H_2O]
814.4952	1	1414.3	$C_{42}H_{72}O_{13}$	(M+HCOO)- [-H_2O]

Predicted isotope match table								
Isotope	*m/z*	Calc *m/z*	Diff (ppm)	Calc Abund	Abund %	Calc abund %	Abund sum %	Cal abund sum %
1	811.4843	811.4849	0.0006	41372.4	100	100	60.4	60.6
2	812.4875	812.4883	0.0008	19799.8	46.98	47.86	28.38	29
3	813.4905	813.4912	0.0007	5824	15.15	14.08	9.15	8.53
4	814.4952	814.494	-0.0012	1275.9	3.43	3.08	2.07	1.87

Figure S10. HRESIMS data of Ginsenoside Rg3 (**10**)

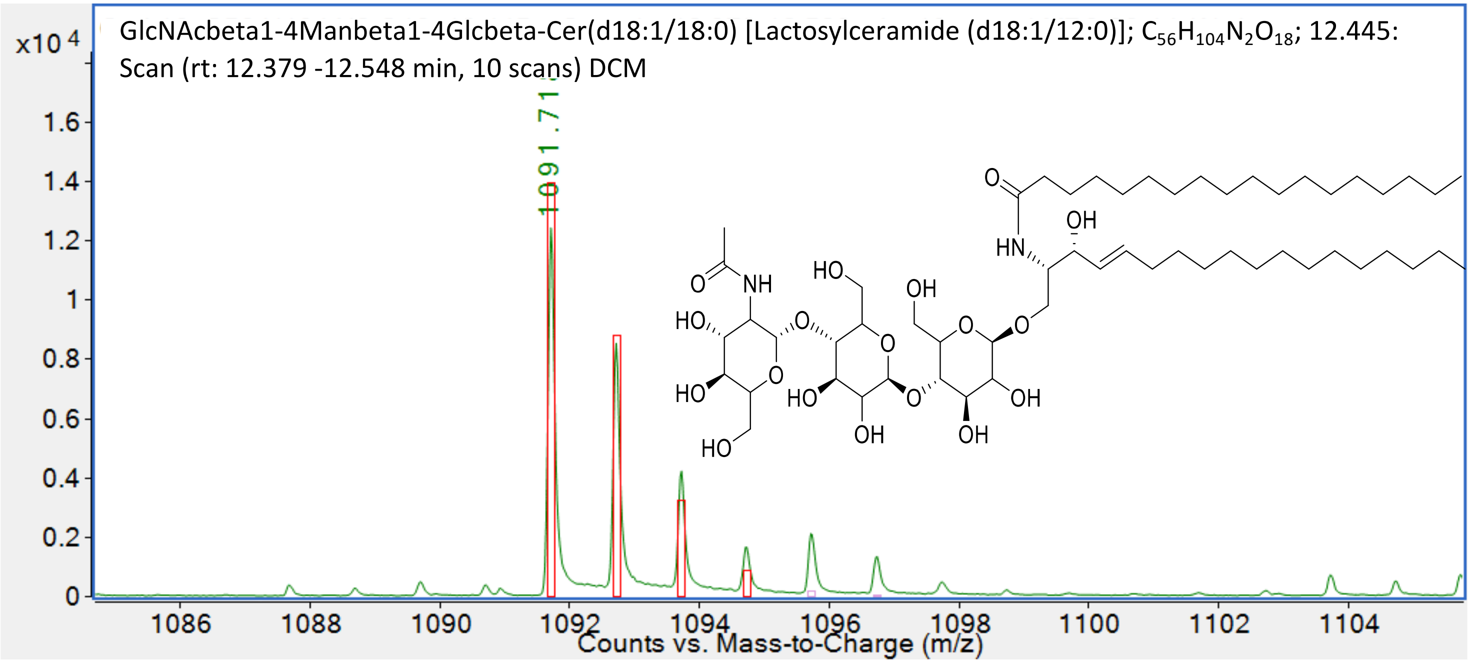

MS spectrum peak list				
m/z	***z***	**Abund**	**Formula**	**Ion**
1091.718	1	12451.4	$C_{56}H_{104}N_2O_{18}$	(M-H)-
1092.7207	1	8555.1	$C_{56}H_{104}N_2O_{18}$	(M-H)-
1093.722	1	4252.3	$C_{56}H_{104}N_2O_{18}$	(M-H)-
1094.7233	1	1693.4	$C_{56}H_{104}N_2O_{18}$	(M-H)-

Predicted isotope match table

Isotope	*m/z*	Calc *m/z*	Diff (ppm)	Calc Abund	Abund %	Calc abund %	Abund sum %	Cal abund sum %
1	1091.718	1091.7211	0.0031	13977.8	100	100	46.2	51.86
2	1092.7207	1092.7245	0.0038	8829.6	68.71	63.17	31.74	32.76
3	1093.722	1093.7274	0.0054	3259.8	34.15	23.32	15.78	12.09
4	1094.7233	1094.7303	0.007	884.9	13.6	6.33	6.28	3.28

Figure S11. HRESIMS data of GlcNAcbeta1-4Manbeta1-4Glcbeta-Cer(d18:1/18:0) [Lactosylceramide (d18:1/12:0)] (**11**)

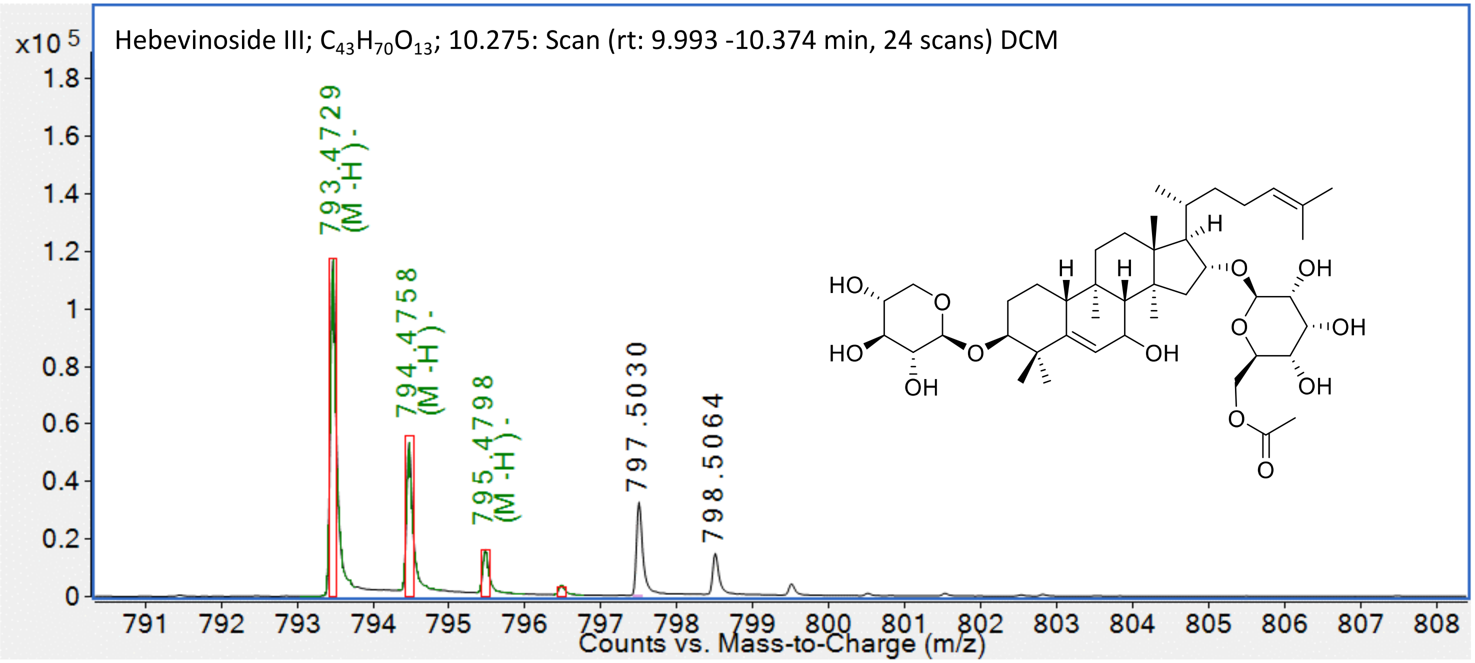

MS spectrum peak list				
m/z	*z*	Abund	Formula	Ion
793.473	1	117372.2	$C_{43}H_{70}O_{13}$	(M-H)-
794.4758	1	53666.6	$C_{43}H_{70}O_{13}$	(M-H)-
795.4799	1	16412.4	$C_{43}H_{70}O_{13}$	(M-H)-
796.4832	1	3380.2	$C_{43}H_{70}O_{13}$	(M-H)-

Predicted isotope match table

Isotope	*m/z*	Calc *m/z*	Diff (ppm)	Calc Abund	Abund %	Calc abund %	Abund sum %	Cal abund sum %
1	793.473	412.3397	0.0014	115925.2	100	100	61.51	60.75
2	794.4758	413.3431	0.002	55408.1	45.72	47.8	28.12	29.04
3	795.4799	414.3459	0.0008	16046.8	13.98	13.84	8.6	8.41
4	796.4832	415.3486	0.0003	3451.4	2.88	2.98	1.77	1.81

Figure S12. HRESIMS data of Hebevinoside III (**12**)

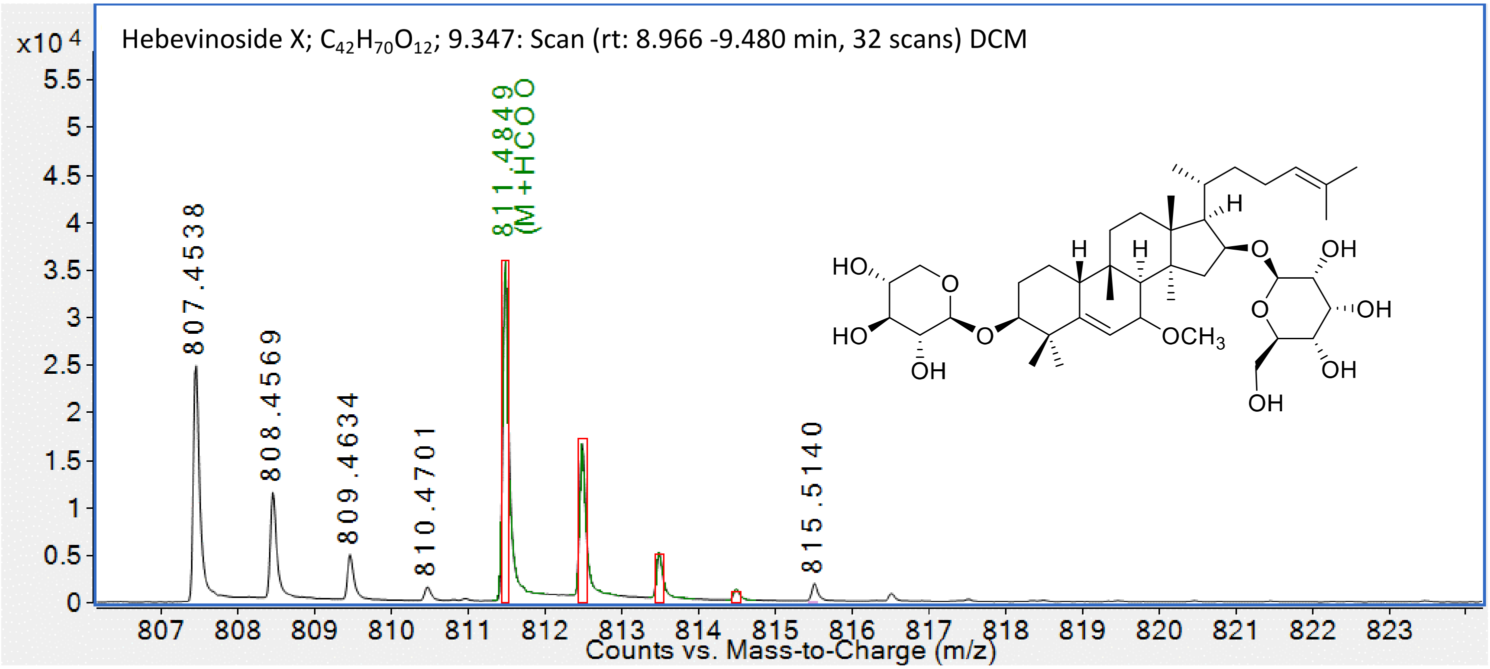

MS spectrum peak list				
m/z	*z*	Abund	Formula	Ion
811.4849	1	36042.9	$C_{41}H_{68}O_{12}$	(M+CH_3COO)-
812.4882	1	16792.4	$C_{41}H_{68}O_{12}$	(M+CH_3COO)-
813.4919	1	5388.4	$C_{41}H_{68}O_{12}$	(M+CH_3COO)-
814.4955	1	1191.5	$C_{41}H_{68}O_{12}$	(M+CH_3COO)-

Predicted isotope match table

Isotope	*m/z*	Calc *m/z*	Diff (ppm)	Calc Abund	Abund %	Calc abund %	Abund sum %	Cal abund sum %
1	811.4849	811.4849	0	36005.2	100	100	60.66	60.6
2	812.4882	812.4883	0	17231.2	46.59	47.86	28.26	29
3	813.4919	813.4912	-0.0007	5068.5	14.95	14.08	9.07	8.53
4	814.4955	814.494	-0.0015	1110.4	3.31	3.08	2.01	1.87

Figure S13. HRESIMS data of Hebevinoside X (**13**)

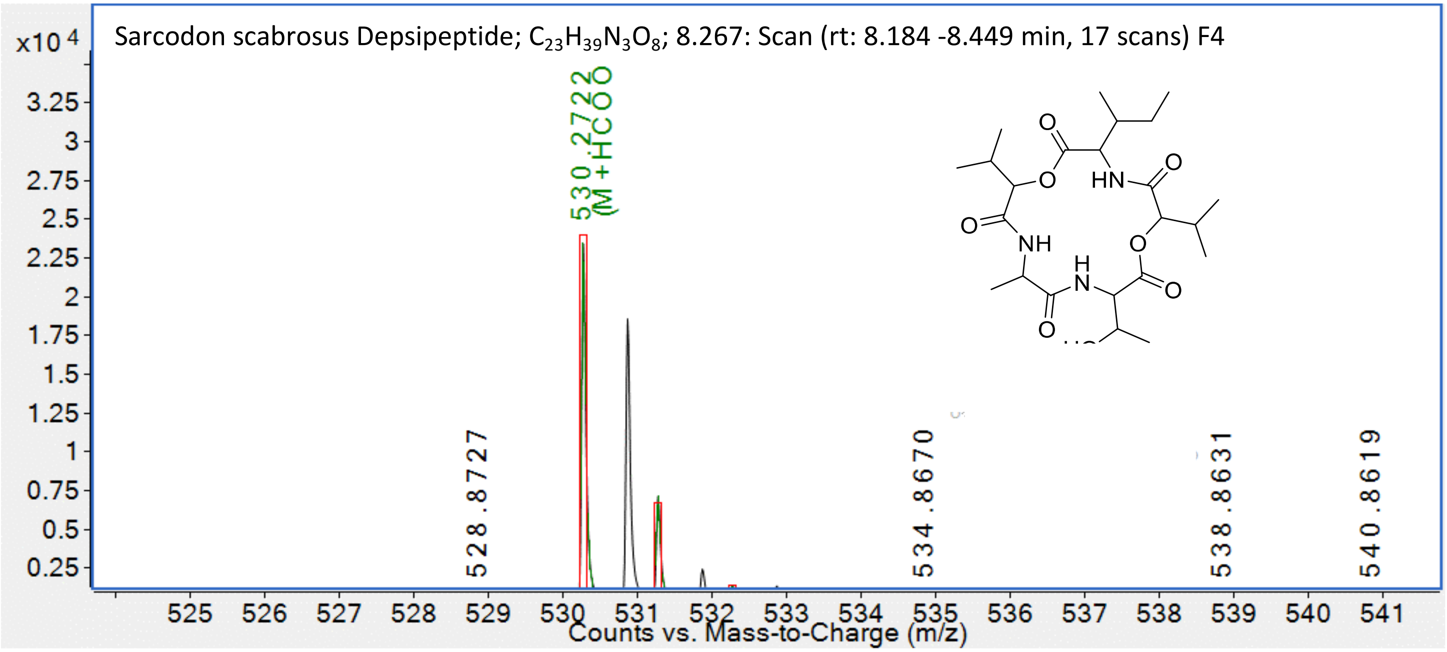

MS spectrum peak list				
m/z	***z***	**Abund**	**Formula**	**Ion**
530.2722	1	23493.6	$C_{23}H_{39}N_3O_8$	(M+HCOO)-
531.2749	1	7144.6	$C_{23}H_{39}N_3O_8$	(M+HCOO)-
532.2775	1	1395.4	$C_{23}H_{39}N_3O_8$	(M+HCOO)-

Predicted isotope match table

Isotope	*m/z*	Calc *m/z*	Diff (ppm)	Calc Abund	Abund %	Calc abund %	Abund sum %	Cal abund sum %
1	530.2722	530.2719	0.0003	23959.7	100	100	73.34	74.8
2	531.2749	531.2751	-0.0002	6683.5	30.41	27.89	22.3	20.86
3	532.2775	532.2775	0	1390.4	5.94	5.8	4.36	4.34

Figure S14. HRESIMS data of Sarcodon scabrosus Depsipeptide (**14**)

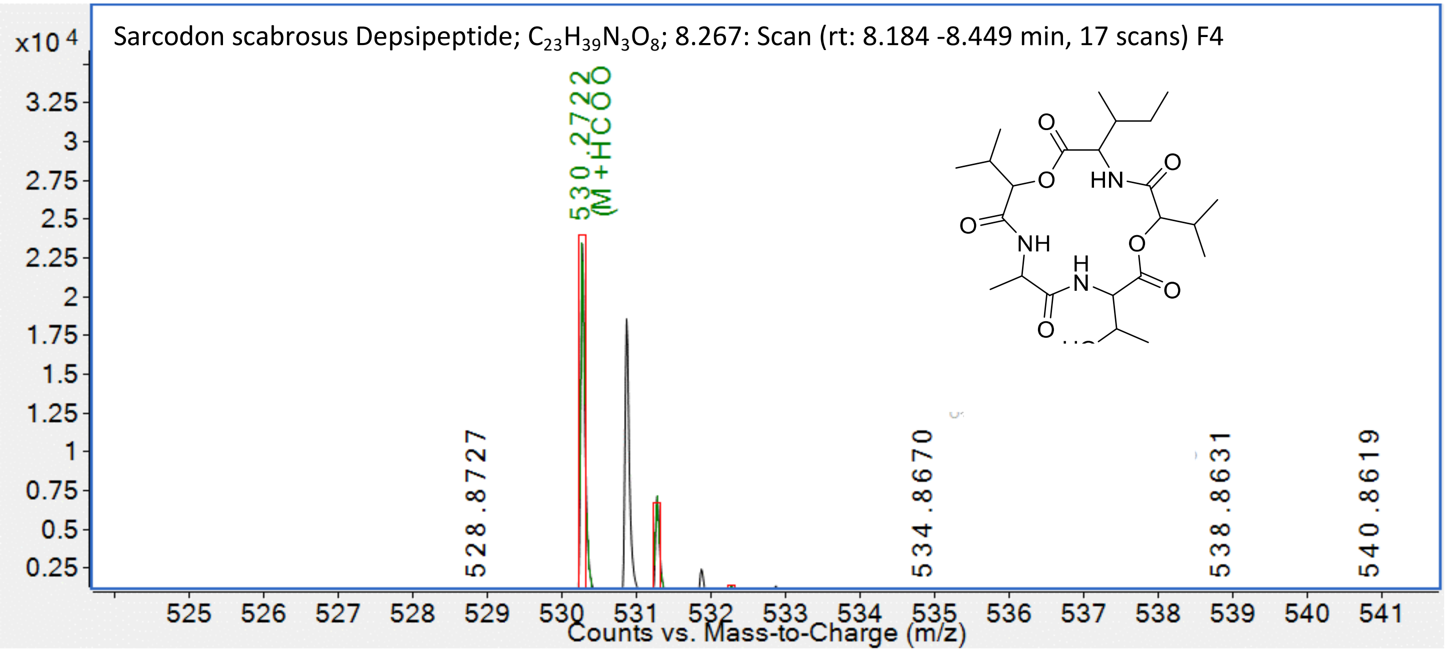

MS spectrum peak list				
m/z	***z***	**Abund**	**Formula**	**Ion**
1115.6568	1	114594.9	$C_{59}H_{92}O_{16}$	$(M+CH_3COO)^-$
1116.6597	1	75801.3	$C_{59}H_{92}O_{16}$	$(M+CH_3COO)^-$
1117.6652	1	38938.1	$C_{59}H_{92}O_{16}$	$(M+CH_3COO)^-$
1118.6692	1	13151.9	$C_{59}H_{92}O_{16}$	$(M+CH_3COO)^-$

Predicted isotope match table

**Isotope**	***m/z***	**Calc *m/z***	**Diff (ppm)**	**Calc Abund**	**Abund %**	**Calc abund %**	**Abund sum %**	**Cal abund sum %**
1	1115.6568	1115.6524	0.0044	120341.2	100	100	47.26	49.63
2	1116.6597	1116.6558	0.0039	81536.3	66.15	67.75	31.26	33.63
3	1117.6652	1117.6589	0.0063	31643.9	33.98	26.3	16.06	13.05
4	1118.6692	1118.6618	0.0074	8964.7	11.48	7.45	5.42	3.7

Figure S15. HRESIMS data of Gambieric acid A (**15**)

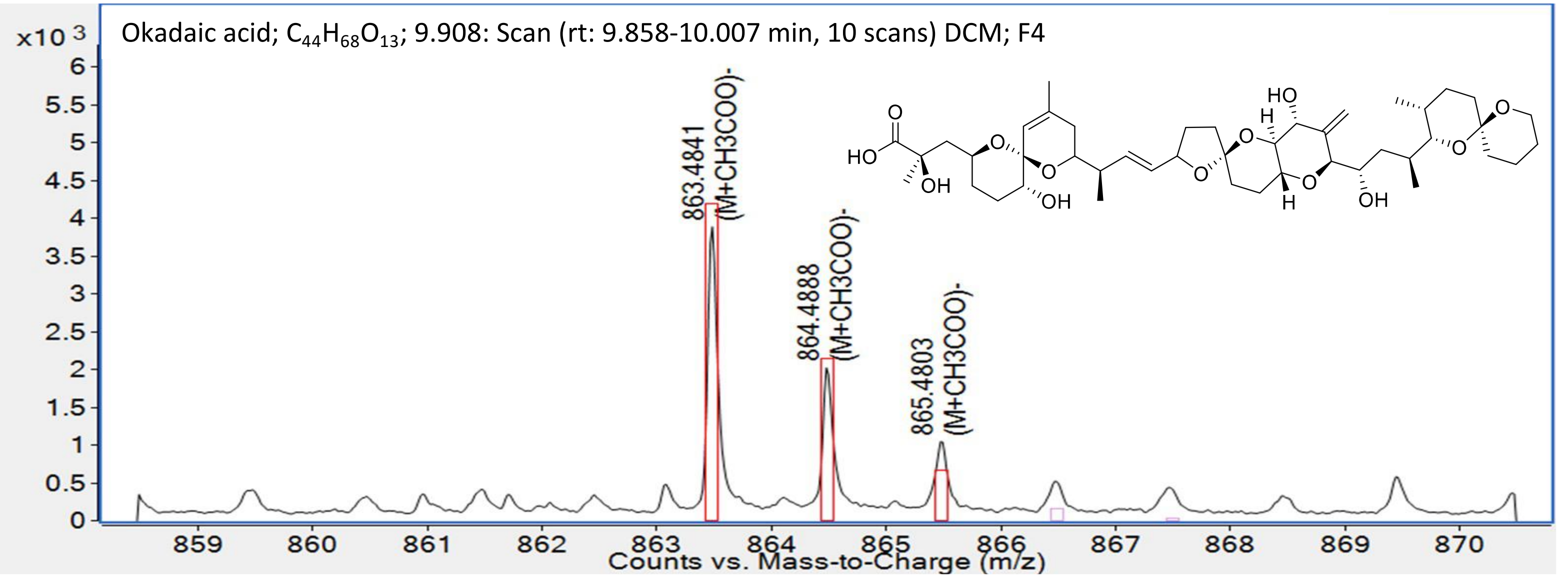

MS spectrum peak list				
m/z	***z***	**Abund**	**Formula**	**Ion**
863.4841	1	3902.4	$C_{44}H_{68}O_{13}$	$(M+CH_3COO)^-$
864.4888	1	2044.1	$C_{44}H_{68}O_{13}$	$(M+CH_3COO)^-$
865.4803	1	1063.5	$C_{44}H_{68}O_{13}$	$(M+CH_3COO)^-$

Predicted isotope match table

Isotope	*m/z*	Calc *m/z*	Diff (ppm)	Calc Abund	Abund %	Calc abund %	Abund sum %	Cal abund sum %
1	863.4841	863.4798	0.0043	4196.9	100	100	55.67	59.87
2	864.4888	864.4833	0.0055	2146.3	52.38	24.85	29.16	30.62
3	865.4803	865.4862	-0.0059	666.9	27.25	3.78	15.17	9.51

Figure S16. HRESIMS data of Okadaic acid (**16**)

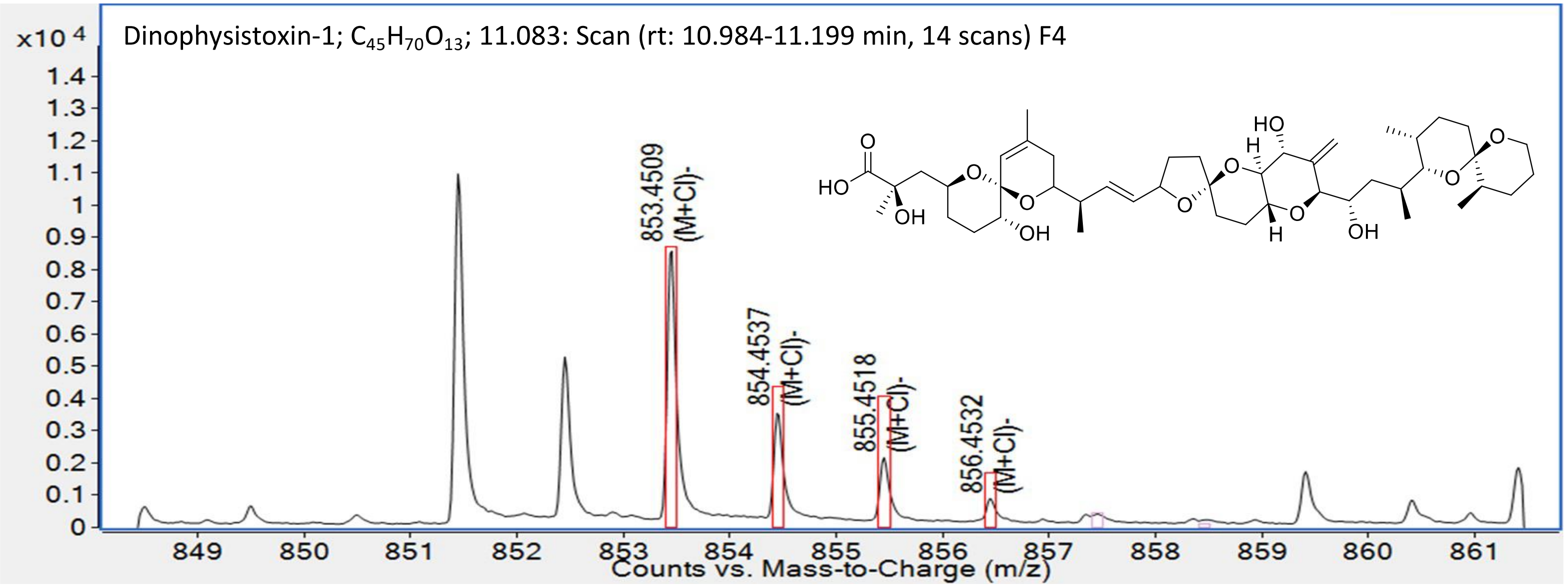

MS spectrum peak list				
m/z	*z*	Abund	Formula	Ion
853.4509	1	8718.9	$C_{45}H_{70}O_{13}$	(M+Cl)-
854.4537	1	3606.6	$C_{45}H_{70}O_{13}$	(M+Cl)-
855.4518	1	2175.5	$C_{45}H_{70}O_{13}$	(M+Cl)-
856.4532	1	911.9	$C_{45}H_{70}O_{13}$	(M+Cl)-

Predicted isotope match table

Isotope	*m/z*	Calc *m/z*	Diff (ppm)	Calc Abund	Abund %	Calc abund %	Abund sum %	Cal abund sum %
1	853.4509	853.451	0.0001	7131.2	100	100	46.27	56.57
2	854.4537	854.4545	0.0008	3563.5	41.37	49.97	23.12	23.4
3	855.4518	855.4511	-0.0007	3343.8	24.95	46.89	21.69	14.11
4	856.4532	856.453	-0.0002	1374.4	10.46	19.27	8.92	5.92

Figure S17. HRESIMS data of Dinophysistoxin-1 (**17**)

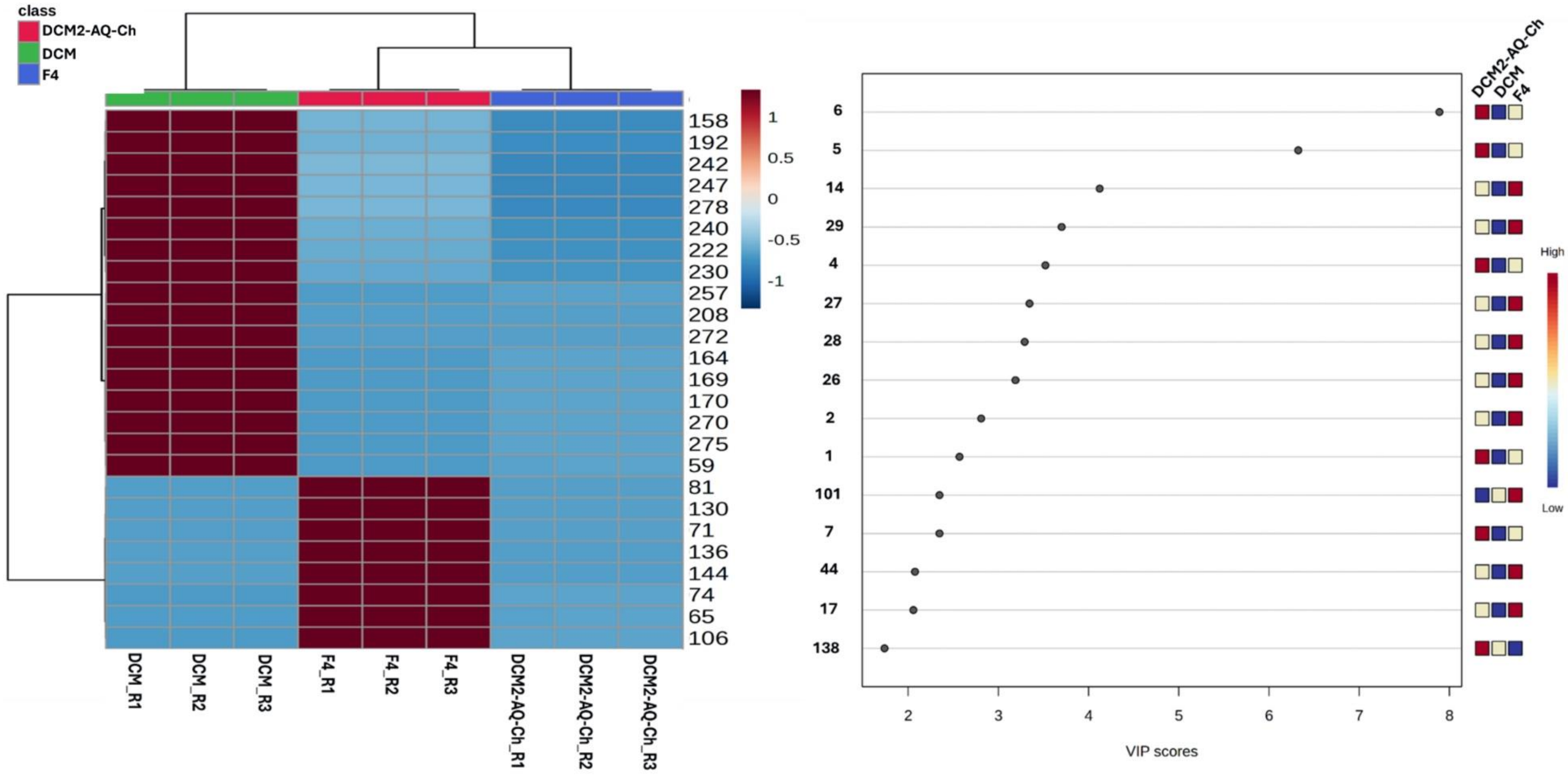

Figure S18. Metabolic **Profiling Across Classes: Heatmap, VIP Scores Analysis of Key Compounds from *Coolia malayensis*. A)** Heat map. The colored areas on the map correspond to the concentrations of different compounds found in DCM, DCM2-AQ-Ch, and F4. Each row represents a compound, and each column represents the repetitions of each analyzed sample. Red indicates high concentrations, while blue indicates low concentrations. **B)** PLS-DA assigns VIP scores to the compounds found in DCM2-AQ-Ch, DCM, and F4. The scores, ranging from low to high, determine the importance of the variables. The colored boxes on the right represent the relative concentration of each compound. High levels of red indicate high concentrations, while low levels of blue indicate low concentrations.
